# Specific Zn(II)-binding site in the C-terminus of Aspf2, a zincophore from *Aspergillus fumigatus*

**DOI:** 10.1093/mtomcs/mfac042

**Published:** 2022-06-14

**Authors:** Kinga Garstka, Aleksandra Hecel, Henryk Kozłowski, Magdalena Rowińska-Żyrek

**Affiliations:** Faculty of Chemistry, University of Wrocław, F. Joliot-Curie 14, 50-383 Wrocław, Poland; Faculty of Chemistry, University of Wrocław, F. Joliot-Curie 14, 50-383 Wrocław, Poland; Faculty of Chemistry, University of Wrocław, F. Joliot-Curie 14, 50-383 Wrocław, Poland; Institute of Health Sciences, University of Opole, Katowicka 68 St, 45-060 Opole, Poland; Faculty of Chemistry, University of Wrocław, F. Joliot-Curie 14, 50-383 Wrocław, Poland

**Keywords:** zincophore, thermodynamics, potentiometry, Zn(II)- and Ni(II)-binding peptides

## Abstract

*Aspergillus fumigatus*, one of the most widespread opportunistic human fungal pathogens, adapts to zinc limitation by secreting a 310 amino acid Aspf2 zincophore, able to specifically bind Zn(II) and deliver it to a transmembrane zinc transporter, ZrfC. In this work, we focus on the thermodynamics of Zn(II) complexes with unstructured regions of Aspf2; basing on a variety of spectrometric and potentiometric data, we show that the C-terminal part has the highest Zn(II)-binding affinity among the potential binding sites, and Ni(II) does not compete with Zn(II) binding to this region. The 14 amino acid Aspf2 C-terminus coordinates Zn(II) via two Cys thiolates and two His imidazoles and it could be considered as a promising *A. fumigatus* targeting molecule.

## Graphical Abstract

**Graphical Abstract:**
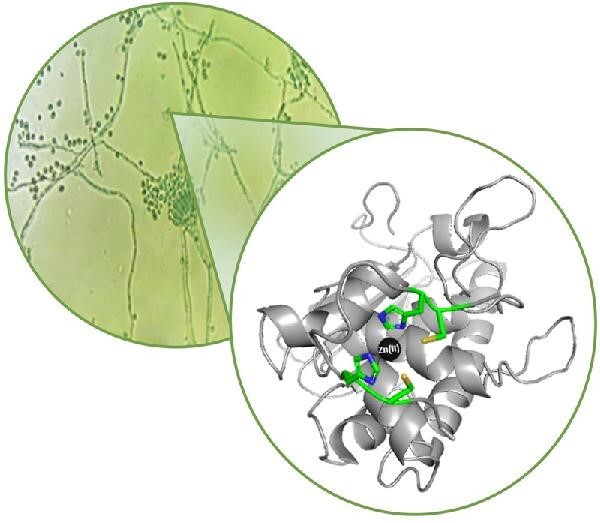
The C-terminal region of the Aspf2 zincophore is its most probable Zn(II) binding site.

Twitter short summary The Aspf2 zincophore from *A. fumigatus* specifically binds Zn(II) via two Cys thiols and two His imidazoles in its unstructured C-terminal region; Ni(II) ions cannot displace Zn(II) from this binding site.

## Introduction

One of the biggest problems with finding new, highly specific antifungal drugs is the fact that fungi share basic metabolic pathways and basic cellular mechanisms with their human hosts. Since both are eukaryotes, the similarity is much greater than in the case of prokaryotic bacteria.^1^ Consequently, in order to design a highly specific antifungal drug, it is necessary to understand the difference in human and fungal metabolism.^2^ One of the significant differences between mammalian and fungal cells that can be aimed at is the mechanism of zinc uptake, which is one of the critical aspects of fungal virulence and survival.^3,4^ Because metals are necessary for many vital cellular functions, fungi have developed highly specified mechanisms of their acquisition and the host developed mechanisms that limit the microbes’ access to the essential metal nutrients, in a process known as “nutritional immunity”.^5^ One of the major differences between the human and fungal metabolism that can be aimed at in potential antifungal therapeutics is the zincophore-based Zn(II) transport—understanding its molecular basis, thermodynamics, and coordination chemistry is crucial for improving the therapy and diagnostics of fungal infections.^5,6^

The constant increase of drug resistance against antibiotics and antifungal agents is a heavy burden on health systems and prompts reconsidering common therapies and availability of innovative drugs. Aspergillosis is one of the most common fungal infections and the number of species known as *Aspergillus* is more than 800.^7,8^ However, only four of them (*Aspergillus fumigatus, Aspergillus flavus, Aspergillus terrus*, and *Aspergillus niger*) are responsible for causing almost 90% of all human *Aspergillus* infections.^7^ The most clinically significant species is *A. fumigatus, which* causes more than half of aspergillosis cases in the USA with a 50–95% mortality rate.^9,10^ It is a saprophytic fungus, which produces a very large amount of conidia and is one of the most widespread airborne fungal pathogens.^10,11^  *Aspergillus fumigatus* can cause several types of disease, including invasive pulmonary aspergillosis, allergic bronchopulmonary aspergillosis, or chronic pulmonary aspergillosis not only in immunosuppressed patients, but also in those with underlying illnesses such chronic obstructive pulmonary disease or tuberculosis and in patients with healthy immune systems.^12–15^ Thus, while the disease only develops in a small fraction of patients who have problems with the immune system or lung pathology, daily inhalation of *Aspergillus* spores is very common and causes the disease to develop more often for very simple biological reasons: *A. fumigatus* is present in high concentrations in the atmosphere, grows faster than any other airborne fungi at 40°C, and it can develop specific systems that enable host colonization.^13,14^ Given the increasing drug resistance and growing number of patients at risk, this pathogen has become a serious clinical and financial burden for global healthcare systems, comparable to the problem of “superbug” infections.^7,16,17^ Therefore, the need to introduce novel, highly specific antifungal treatments is undeniable.

Zinc is an essential metal for a plethora of cellular events such as growth, development, or reactive oxygen species detoxification, but also for amino acid metabolism, nitrogen utilization, or cell division.^3,15,18,19^ Free, nonprotein-bound zinc concentration in host cells is as low as sub-nanomolar and therefore its acquisition by fungal cells is a serious challenge.^20^  *Aspergillus fumigatus* has evolved several mechanisms to overcome the host nutritional immunity by expressing zinc importers as well as exporters to balance its zinc requirements in Zn(II) overload and limitation.^21^ Also Ni(II) is a necessary nutrient for *A. fumigatus*, crucial for the proper functioning of hydrogenase.^22^ Furthermore, since its coordination requirements may be similar to that of Zn(II), the two metals can compete with each other for the same binding sites.

**Fig. 1.**
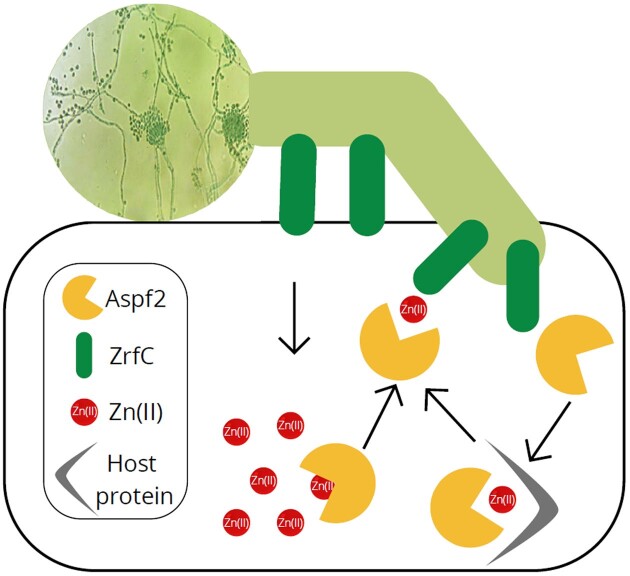
Schematic model of *Aspergillus fumigatus* zinc scavenging from host cells. After invasion of the host cell, Aspf2 is expressed and secreted. It binds zinc, either in the form of free Zn(II) ions or from zinc-binding proteins of the host. Reassociation with the *A. fumigatus* cell surface and Zn(II) transportation into the cell occurs via a Aspf2–ZrfC interaction.^15^

The expression of the *Aspergillus fumigatus* the Aspf2 and ZrfC zinc transporter-encoding genes is regulated by both pH and Zn(II) concentration—they are transcribed at higher levels and are required for fungal growth under acidic pH values.^23^ In neutral and alkaline zinc-limiting media, the dominating zinc-uptake system relies solely on the ZrfC transporter.^24^ In slightly acidic environments, *A. fumigatus* secretes the Aspf2 zincophore, a 310 amino acid protein that is able to sequester zinc from the host. Aspf2 is released to the surrounding host cells, where it binds the host's zinc (both free and that bound to other biomolecules) and then returns to the fungal cell and delivers the metal to the fungal pathogen through a physical interaction with the ZrfC transporter (Fig. 1).^25^

Aspf2 and zrfc genes are encoded at the same locus, have the same promoter, and are quite well conserved in several other fungal species, e.g. *Candida albicans*, where pra1 and zrt1 are orthologues of *A. fumigatus* aspf2 and zrfc.^15^ The two zincophores—Aspf2 from *A. fumigatus* and Pra1 from *C. albicans* share 43% identity and possess similar zinc-binding motifs. Moreover, the Zn(II) transporters ZrfC from *A. fumigatus* and Zrt1 from *C. albicans* share 48% identity, giving the basis for a hypothesis that this zinc-binding transport is evolutionally conserved and works in a similar way.^26^ From the functional point of view, both the Aspf2/ZrfC and Pra1/Zrt1 zinc-uptake systems are analogous to bacterial zinc ABC transporters, where the bacteria secrete the zinc scavenger—ZnuA, which delivers Zn(II) to the transmembrane protein—ZnuB.^27^

The purpose of this work is to understand the interactions of Zn(II) with Aspf2, the *A. fumigatus* zincophore, in order to find the site that has the highest affinity toward Zn(II). Our previous studies with *C. albicans* Pra1 zincophore pointed out the C-terminal site as the one with the highest affinity toward this metal; this site binds zinc ions through a set of four histidine imidazoles (His288, His290, His292, and His298) and such a complex is most likely selectively recognized by the Zrt1 transporter.^6,15^ The most probable Zn(II)-binding site on the Zrt1 transporter is the KKCHFHAGVEHCVDDNNHDA region [residues from 151 to 170; three imidazoles and one thiolate (His156, His161, His168, and Cys162) participate in the coordination)].^6^ The Zn(II)–Zrt1 binding is more stable that the Zn(II)–Pra1 one and this phenomenon allows for the Zn(II) transfer from Pra1 zincophore to the Zrt1 transporter.^6^

**Fig. 2.**
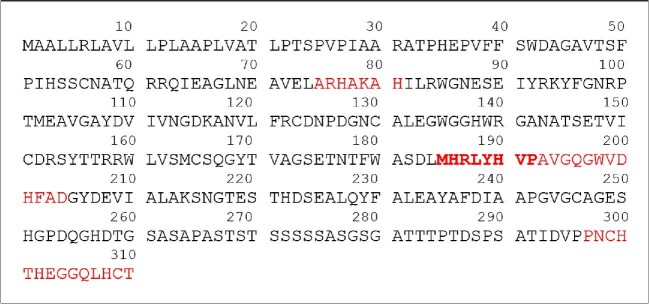
Amino acid sequence of Aspf2; probable Zn(II)-binding sites are highlighted in red.

In this work, we focus on four Aspf2 regions, which are the most probable Zn(II)-binding sites. They are chosen on the basis of three criteria: (i) having probable zinc-binding residues, (ii) being unstructured, and (iii) being evolutionally conserved sites. The structure predicted by Phyre2 (a remote homology recognition technique, capable of generating reliable protein models^28^ shows that the chosen Aspf2 regions are unstructured and therefore appropriate for our peptide-based approach; they contain probable Zn(II)-binding sites and are all well conserved [_75_ARHAKAH_81, 185_MHRLYHVP_192, 185_MHRLYHVPAVGQGWVDHFAD_204_, and _297_PNCHTHEGGQLHCT_310_ (highlighted in red in Fig. 2)]. It is important to keep in mind that in the full-length peptide, the metal-binding sites might also occur in locally structured regions.

We check the thermodynamic parameters and precise Zn(II)- and Ni(II)-binding sites, keeping in mind that Ni(II) is a biologically relevant metal ion, essential for *C. albicans* hydrogenase,^29^ and could theoretically complete with Zn(II) for the binding sites in its zincophore.

## Experimental

### Materials

All peptides, Ac–MHRLYHVP–NH_2_, Ac–MHRLYHVPAVGQGWVDHFAD–NH_2_, Ac–ARHAKAH–NH_2_, and Ac–PNCHTHEGGQLHCT, were purchased from Karebay Biochem (certified purity: 98%) and were used as received. The carbonate-free stock solution of 0.1 M KOH was purchased from Sigma-Aldrich and then potentiometrically standardized with potassium hydrogen phthalate. Zn(II) and Ni(II) chlorides were extra-pure products (Sigma-Aldrich). The concentrations of stock solutions of these salts were determined by inductively coupled plasma mass spectrometry.

### Mass spectrometric measurements

High-resolution mass spectra were obtained on a Bruker FT-ICR spectrometer (Bruker Daltonik, Bremen, Germany), equipped with an Apollo II electrospray ionization source with an ion funnel. The mass spectrometer was operated in the positive ion mode. The instrumental parameters were as follows: scan range: m/z 100–2000, dry gas: nitrogen, temperature: 453 K, and ion energy: 5 eV. The capillary voltage was optimized to the highest S/N ratio and it was 4800 V. The small changes of voltage (±500 V) did not significantly affect the optimized spectra. The samples [metal: ligand in a 1:1 stoichiometry, (ligand)tot = 10^−4^ M] were prepared in a 1:1 acetonitrile–water mixture. The variation of the solvent composition down to 5% of acetonitrile did not change the species composition. The sample was infused at a flow rate of 3 μL min^−1^. The instrument was calibrated externally with the Tunemix™ mixture (Bruker Daltonik) in quadratic regression mode. Data were processed using the Bruker Compass DataAnalysis 4.0 program. The mass accuracy for the calibration was better than 5 ppm, enabling together with the true isotopic pattern (using SigmaFit) an unambiguous confirmation of the elemental composition of the obtained complex.

### Potentiometric measurements

The stability constants for all ligands and their Zn(II) and Ni(II) complexes were calculated from titration curves carried out over the pH range of 2–11 at 298 K. The ionic strength 0.1 M NaClO_4_ was used for Ac–ARHAKAH–NH_2_ and Ac–PNCHTHEGGQLHCT; in the case of Ac–MHRLYHVP–NH_2_ and Ac–MHRLYHVPAVGGWVDHFAD–NH_2_, 0.04 M Sodium dodecyl sulfate was used to improve the solubility of the peptides. The total volume of the solution used was 2.5 mL. The potentiometric titrations were performed using a Dosimat 665 Metrohmititrator connected to a Methrom 691 pH meter and a Methrom LL Unicode glass electrode or a Mettler Toledo pH inLab Science electrode. The thermostabilized glass cell was equipped with a magnetic stirring system, a microburet delivery tube, and an inlet–outlet tube for argon. Solutions were titrated with 0.1 M carbonate-free KOH. The electrodes were calibrated daily for hydrogen ion concentration by titrating HClO_4_ with KOH using a total volume of 3 mL. The purities and the exact concentrations of the ligand solutions were determined by the Gran method. The ligand concentration was 0.5 mM and the Zn(II) and Ni(II) to ligand ratio was 1:1. The HYPERQUAD 2006 program was used for the stability constant calculations. The standard deviations were computed by using HYPERQUAD 2006 and referenced to random errors only.^30^ The constants for hydrolytic Zn(II) and Ni(II) species were used in these calculations. The speciation and competition diagrams were computed using the HYSS program.^31^

### Spectroscopic studies

The absorption spectra were recorded on a Jasco-730 spectrophotometer, in the range 200–800 nm, using a quartz cuvette with an optical path of 1 cm. Circular dichroism (CD) spectra were recorded on a Jasco J-1500 CD spectrometer in the 200–800 nm range, using a quartz cuvette with an optical path of 1 cm in the visible and near-UV range. To determine the secondary structure of the analysed samples, measurements were made in the 180–270 nm, using a quartz cuvette with an optical path of 0.01 cm. The concentration of sample solutions used for spectroscopic studies was similar to those employed in the potentiometric experiment. The metal: ligand ratio was 1:1. All spectroscopic measurements were recorded in the pH range 3–11. The pH of the samples was adjusted with the appropriate amounts of HClO_4_ and NaOH solutions. The UV–Vis and CD spectroscopy parameters were calculated from the spectra obtained at the pH values corresponding to the maximum concentration of each particular species, based on distribution diagrams. OrginPro 2016 was used to process and visualize the obtained spectra.

NMR spectra were recorded at 14.1 T on Bruker Avance III 600 MHz equipped with a Silicon Graphics workstation. The temperatures were controlled with an accuracy of ±0.1 K. Suppression of the residual water signal was achieved by excitation sculpting, using a selective square pulse on water 2 ms long. The samples were prepared in 100% D_2_O (99.95% from Merck) and in a 90% H_2_O and 10% D_2_O mixture. The proton resonance assignment was accomplished by 2D ^1^H–^1^H total correlation spectroscopy (TOCSY) and nuclear Overhauser effect spectroscopy experiments, carried out with standard pulse sequences. Samples of the analysed complexes were prepared by adding Zn(II) (in the M(II)/L molar ratio 1:1) and Ni(II) (in the M(II)/L molar ratio 1:10 (due to its possibly paramagnetic nature) to an acidic solution of a 1 mM ligand (pH 3) and the pH was then increased to 7.4. The NMR spectra recorded in the presence and in the absence of metal ions pointed out precise metal-binding sites and the combination of all used methods allowed to explain the coordination geometries. Total processing and analysis were performed using Brucker TOPSPIN 2.1 and Sparky programs.^32^

## Results and discussion

Mass spectrometry confirmed that the tested peptides are capable of forming Ni(II) and Zn(II) complexes in a 1:1 (metal: ligand ratio) stoichiometry. The results are presented in the Supplementary Figs. S1 and 2, ESI†.

### Ligand protonation

The Ac–ARHAKAH–NH_2_ peptide acts like a typical LH_4_ acid. The first two pK_a_ values (5.85, 6.72) come from the deprotonation of two imidazole groups of the histidine residues. The next constant (9.79) corresponds to the deprotonation of the lysine side chain group and the highest one (11.14) to the deprotonation of arginine. The results are reported in Table 1.

**Table 1:** Potentiometric data for the proton, Zn(II) and Ni(II) complexes of the, Ac–ARHAKAH–NH_2_, Ac–MHRLYHVP–NH_2_, Ac–MHRLYHVPAVGQGWVDHFAD–NH_2_ and Ac–PNCHTHEGGQLHCT

	Ac–ARHAKAH–NH_2_			Ac–MHRLYHVP–NH_2_			Ac–MHRLYHVPAVGQG WVDHFAD–NH_2_			Ac–PNCHTHEGGQLHCT		
Species	logβ	pK_a_		logβ			logβ	pK_a_		logβ	pK_a_	
HL	11.14	11.14	(R)	11.21	11.21	(R)	11.24	11.24	(R)	9.49	9.49	(C)
H_2_L	20.93	9.79	(K)	20.77	9.56	(Y)	21.36	10.12	(Y)	18.49	9.00	(C)
H_3_L	27.65	6.72	(H)	29.10	8.33	(H)	29.96	8.60	(H)	25.72	7.23	(H)
H_4_L	33.50	5.85	(H)	36.32	7.22	(H)	37.84	7.88	(H)	32.26	6.54	(H)
H_5_L							44.18	6.34	(H)	37.99	5.73	(H)
H_6_L							48.90	4.72	(D)	42.23	4.24	(E)
H_7_L										45.31	3.08	(C_term_)
Zn(II) complexes												
ZnH_5_L							47.01					
ZnH_4_L							41.57	5.44	(H)			
ZnH_3_L							34.45	7.12	(H)	30.74		
ZnH_2_L	24.45			25.32			27.29	7.16	(H)	25.39	5.35	(H)
ZnHL				16.55	8.77	(H_2_O)	17.73	9.56	(Y)	19.83	5.56	(H)
ZnL	8.65			7.65	8.90	(H_2_O)				12.51	7.32	(C)
ZnH_–1_L	1.03	7.62	(H_2_O)							2.69	9.82	(H_2_O)
ZnH_–2_L	−8.72	9.75	(K)	−11.73								
ZnH_–3_L	−20.51	11.79	(R)									
Ni(II) complexes												
NiH_4_L							41.49			36.28		
NiH_3_L				34.41			34.99	6.50	(H)			
NiH_2_L	24.76			27.17	7.24	(H)	27.72	7.27	(H)	25.11		
NiHL				19.78	7.39	(am)	16.62	11.1	(am)	17.98	7.13	(C)
NiL	7.64			10.39	9.39	(am)				10.55	7.43	(C)
NiH_–1_L	−0.98	8.62	(am)	0.61	9.78	(am)	−0.81			1.63	8.92	(am)
NiH_–2_L	−10.85	9.87	(K)							−8.53	10.16	(am)
NiH_–3_L	−22.23	11.38	(R)	−19.78			−22.49			−19.04	10.51	(am)

The Ac–MHRLYHVP–NH_2_ peptide has four protonation constants. The first two (7.22, 8.33) can be assigned to the deprotonation of two histidine imidazole groups. The next constant, with a pK_a_ of 9.56 is related to the deprotonation of tyrosine, and the most basic one (11.21) corresponds to the deprotonation of arginine.

The Ac–MHRLYHVPAVGQGWVDHFAD–NH_2_ peptide behaves as an LH_6_ acid with the deprotonating groups corresponding to one glutamic acid side chain group (4.72), three histidine imidazole groups (6.34, 7.88, 8.60), tyrosine (10.12), and arginine (11.24).

In the Ac–PNCHTHEGGQLHCT peptide, potentiometric measurements were able to detect seven protonation constants. The first pK_a_ value (3.08) comes from the deprotonation of the C-terminal carboxylic group, and the next one corresponds to the deprotonation of the carboxylic side chain of Glu (pK_a_ = 4.24). The next three constants (5.73, 6.54, 7.23) are related to the deprotonation of three His imidazole groups and the last two (9.00, 9.49) correspond to the deprotonation of two cysteine side chains.

### Zinc complexes

In the case of Ac–ARHAKAH–NH_2_, the first observed complex, ZnH_2_L, with a maximum at pH 7, involves two histidine residues in binding. The loss of the next two protons leads to the ZnL form and is most likely related to the deprotonation of subsequent water molecule bound to the central zinc ion. In the ZnH–_1_L complex, another water molecule bound to the central zinc ion loses a proton. ZnH–_2_L deprotonation comes from a lysine side chain, which does not take part in the coordination and the last form (ZnH–_3_L) corresponds to the deprotonation of the arginine side chain, which also does not participate in binding. The results are reported in Table 1 and Supplementary Fig. S3A, ESI†.

The first observed Zn(II) complex of Ac–MHRLYHVP–NH_2_, ZnH_2_L, has a maximum at around pH 8 and involves two histidine residues in binding. The loss of the next two protons, leading to ZnHL and ZnL forms, is most likely related to the deprotonation of two water molecules bound to the central zinc ion. At higher pH values, the coordination mode does not change and the loss of the next two protons is related to the deprotonation of the tyrosine and arginine side chains, which do not participate in binding (Table 1 and Supplementary Fig. S3B, ESI†).

The first complex observed for Ac–MHRLYHVPAVGQGWVDHFAD–NH_2_ is ZnH_5_L, with a maximum at pH around 5; here, most likely the Zn(II) ion is anchored to one of the aspartic acid side chains. The next three forms, ZnH_4_L, ZnH_3_L and ZnH_2_L, are related to the coordination of subsequent histidine residues. A significant decrease of the pK_a_ values (Table 1) suggests that all histidine are involved in Zn(II) coordination. The last observed form, ZnHL, corresponds to the deprotonation of the tyrosine side chain, which does not participate in binding (Supplementary Fig. S3C, ESI†). The deprotonation of arginine in the complex form lies beyond the measuring scope of the electrode.

**Fig. 3.**
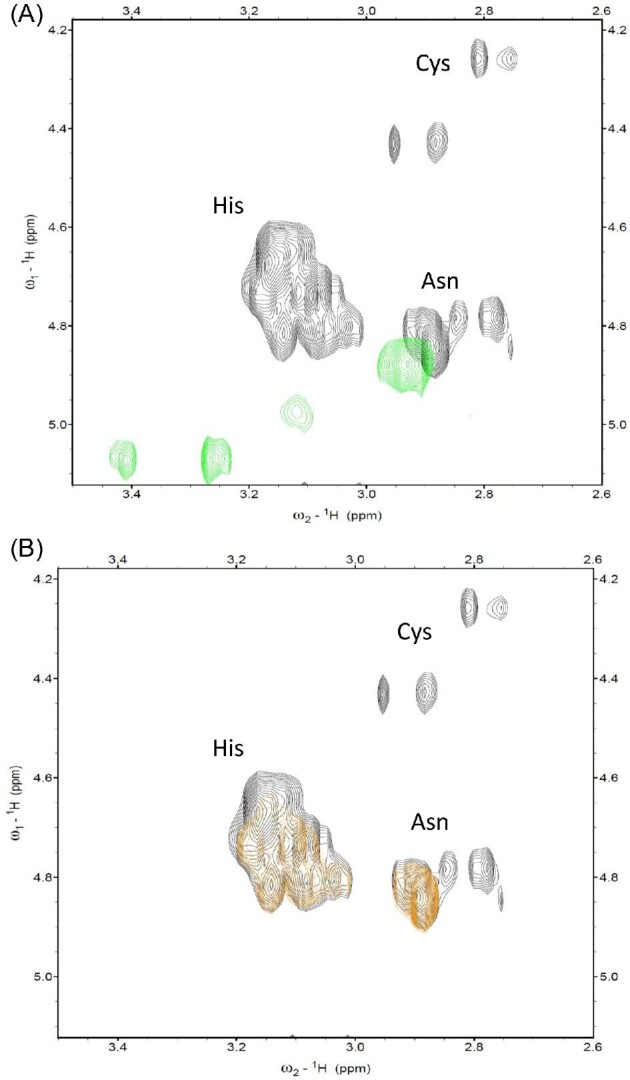
Total correlation spectroscopy (TOCSY) spectra of 3 mM Ac–PNCHTHEGGQLHCT Aspf2 fragment pH 7.4, in the absence (black) and presence of (A) 1 Zn(II) equivalent (green) and (B) 0.1 Ni(II) equivalents (orange).

**Fig. 4.**
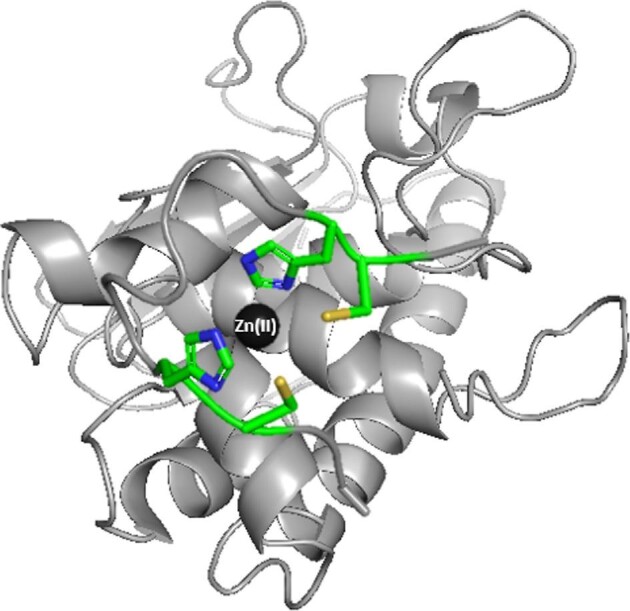
Proposed binding mode for the Zn(II)-Ac–PNCHTHEGGQLHCT C-terminal fragment of Aspf2 zincophore at pH 7.4—a schematic model. Aspf2 structure is based on coordinates simulated by Phyre2 and the figure was generated using PyMOL.^28,39^

Zinc coordination to Ac–PNCHTHEGGQLHCT starts already below pH 5 and the first complex that can be detected is ZnH_3_L. Most probably, in this form, one imidazole of histidine and one cysteine thiolate already participate in binding. In the next form, ZnH_2_L, with a maximum at pH 6.5, the second imidazole nitrogen takes part in binding, judging by the complex pK_a_ value (5.35), which is lower than the one for the free ligand (6.54). The loss of the next proton leads to the ZnHL form, with the maximum at pH 7. A significant decrease of the pK_a_ value calculated for the complexed (5.56) and the free imidazole (7.23) suggests that this histidine is also involved in Zn(II) binding. In the next form, ZnL, the lowered pK_a_ value for the cysteine residue in the complexed ligand compared to the free one suggests that also this residue is bound, and the coordination mode at this point is {2S^−^, 2N_im_} (Fig. 4). This is further confirmed by UV–Vis spectroscopy at pH 7.4, where a pronounced band around 225 nm appears, which can be assigned to a S^–^→Zn(II) charge transfer transition (Supplementary Fig. S4, ESI†).^33^ These results are in good agreement with the NMR data (Fig. 3A and Supplementary Fig. S5A, ESI†), which show that, after the addition of an equimolar amount of Zn(II) to the ligand, both signals assigned to the H_α_–H_β_ correlations of cysteines broaden to baseline, which strongly suggests their involvement in binding. After the addition of Zn(II), we observe shifts in the positions of signals from residues which do not directly participate in binding—the correlations of Thr H_β_–H_γ_, Leu H_α_–H_β_ and H_α_–H_γ_, Asp H_α_–H_β_ and Pro H_α_–H_β_ and H_δ_–H_β_. The resonances of these residues are affected because they sense a different chemical environment after the metal binding to histidine residues, which are in their close proximity. The most basic form, ZnH–_1_L, is related to the deprotonation of a water molecule. The results are reported in Tables 1 and 2, Supplementary Fig. S3D, ESI†.

**Table 2.** Summary of proposed Zn(II)- and Ni(II)-binding models for investigated fragments of Aspf2 zincophore at physiological pH (pH = 7.4)

Ligand	Zn(II) binding mode	Ni(II) binding mode
Ac–ARHAKAH–NH_2_	{2N_im_}	{2N_im_}
Ac–MHRLYHVP–NH_2_	{2N_im_}	{2N_im_}
Ac–MHRLYHVPAVGQGWVDHFAD–NH_2_	{3N_im_, COO^–^}	{3N_im_}
Ac–PNCHTHEGGQLHCT	{2S^–^, 2N_im_}	{2S^–^, 2N_im_}

Please note that in all cases, an equilibrium of different species is present in solution; the coordination modes of the most abundant ones are shown in the table.

### Nickel complexes

The first nickel complex observed at acidic pH for Ac–ARHAKAH–NH_2_ is NiH_2_L, with a maximum at pH 7.5 (Supplementary Fig. S6A, ESI†) and here, most likely, the Ni(II) ion is anchored to two imidazole groups of histidine. The next form, NiL, dominates in the solution around pH 8.7 and engages an amide nitrogen in metal coordination. CD spectra (Supplementary Fig. S7A, ESI†) confirm this finding with pronounced d–d bands at 430 and 520 nm, typical of amide involving square–planar nickel–peptide complexes and a UV–Vis charge transfer band near 275 nm, which can be assigned to N_im_→Ni^2+^ and N^–^→Ni^2+^ charge transfer transitions (Supplementary Fig. S8A, ESI†).^34^ The loss of the next proton leads to the NiH–_1_L form, with a maximum at pH 9, and it is associated with the deprotonation of a second amide nitrogen; the earlier mentioned CD bands increase their intensity. Eventually, at basic pH, a square–planar nickel complex is formed, in which Ni(II) is bound to one histidine imidazole and three amide nitrogens, in a {N_im_, 3N^–^} binding mode; one of the imidazoles coordinated at acidic pH was replaced with an amide. In the NiH–_2_L form, the comparable pK_a_ value of the lysine group in the complexed ligand (pK_a_ = 9.87) with respect to the free one (pK_a_ = 9.79), (Table 1), strongly suggests that this group is not involved in binding. The last complex form, NiH–_3_L, which appears above pH 10, comes from the deprotonation of arginine residue, which also does not participate in binding (complexed ligand—pK_a_ = 11.38, free one—pK_a_ = 11.14).

Six complex forms with Ni(II) for the Ac–MHRLYHVP–NH_2_ peptide were detected (Supplementary Fig. S6B, ESI†). In the NiH_3_L species, the Ni(II) ion is anchored to one of the histidine imidazoles. The loss of the next proton leads to the formation of the NiH_2_L form and the lowered pK_a_ value of the histidine group in the complexed ligand (pK_a_ = 7.24) with respect to the free one (pK_a_ = 8.33), strongly suggests that the second histidine is involved in binding. The NiHL form dominates in the solution at pH around 8.5 and it is associated with the coordination of an amide nitrogen, as indicated by UV–Vis band above pH 10.0 with a maximum at 445 nm (Supplementary Fig. S8B, ESI†). In the next two complexes, NiL and NiH–_1_L, two further amides are involved in Ni(II) binding [and replaced one of the imidazoles in the coordination sphere of Ni(II)]; the CD bands with a negative Cotton effect at 430 nm and a positive Cotton effect at 520 nm gradually increase their intensity (Supplementary Fig. S7B, ESI†), indicating that also in this case, at basic pH, a square–planar nickel complex with a {N_im_, 3N^–^} binding mode is formed.^35^ The last complex form, NiH–_3_L, appears above pH 10 and is connected with the deprotonation of the arginine residue, which does not take part in coordination.

The first detected Ni–Ac–MHRLYHVPAVGQGWVDHFAD–NH_2_ complex is NiH_4_L, with a maximum around pH 6 and here, most likely, the Ni(II) ion is anchored to an imidazole of one of the histidine side chains (Supplementary Fig. S6C, ESI†). The loss of one proton leads to the NiH_3_L form, with a maximum at pH 7. The lowered histidine pK_a_ value in the complexed ligand (pK_a_ = 6.50) with respect to the free one [pK_a_ = 7.88, (Table 1)], strongly suggests that this residue is also directly involved in binding. The next form, NiH_2_L, is associated with the coordination of a third histidine residue (complexed ligand—pK_a_ = 7.27, free one—pK_a_ = 8.60), as indicated by the UV–Vis band with a maximum at 275 nm (Supplementary Fig. S8C, ESI†) that can be related to the N_im_→Ni^2+^ charge transfer transition. The increase of pH leads to the NiHL form, with a maximum at pH around 9.4, which is associated with the coordination of an amide nitrogen. Two further amides are bound in the NiH–_1_L form, which appears above pH 10. The maximum absorption of the d–d band with a positive Cotton effect at 515 nm and a negative one at 425 nm in the CD spectra (Supplementary Fig. S7C, ESI†) and the d–d transitions at 445 nm in the UV–Vis spectra (Supplementary Fig. S8C, ESI†) indicate the formation of the square–planar nickel complex that involves one histidine imidazole and three amide nitrogens in metal binding (Supplementary Fig. S7C, ESI†).

**Fig. 5.**
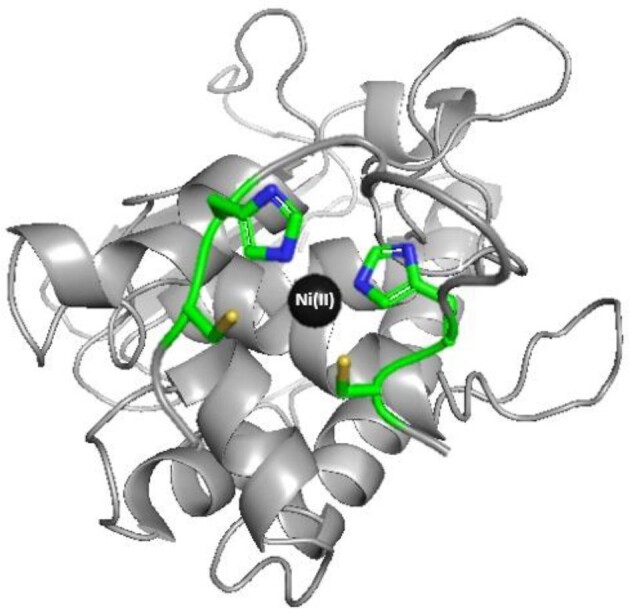
Proposed binding mode for Ni(II)–Ac–PNCHTHEGGQLHCT C-terminal fragment of Aspf2 zincophore at pH 7.4—schematic model. Aspf2 structure is based on coordinates simulated by Phyre2 and the figure was generated using PyMOL^28,39^

Ac–PNCHTHEGGQLHCT starts to bind Ni(II) at pH around 4 and the first detected complex is NiH_4_L, in which Ni(II) is anchored to a histidine imidazole. Two further imidazoles are most probably bound in the NiH_2_L form, with a maximum at around 6.5 (Supplementary Fig. S6D, ESI†). The next complex, NiHL, dominates in solution around pH 7. At this pH, a pronounced CD band at 356 nm appears (Supplementary Fig. S7D, ESI†) that is related to a S^–^→Ni^2+^ transition, which, together with the lowered Cys pK_a_ value with respect to the free ligand (pK_a_ = 7.13 versus pK_a_ = 9.00) indicates thiolate binding to the central Ni(II) ion (Fig. 5).^36^ Loss of the next proton leads to the formation of NiL that dominates in the solution around pH 8 and is related to the deprotonation and coordination of the second cysteine residue, which might replace one of the imidazoles. The lowered pK_a_ value for the cysteine residue in the complexed ligand (7.43) in comparison to the free one (9.49) strongly suggests its participation in binding. NMR data recorded at pH 7.4 further prove this finding, showing a severe broadening of the H_α_–H_β_ correlations of both Cys residues in the presence of Ni(II) (Fig. 3B and Supplementary Fig. S5B, ESI†). In general, square–planar Ni(II)–peptide complexes are yellow and have no free electron on the nickel ion (i.e. the nickel is diamagnetic), whereas octahedral complexes are blue and paramagnetic. At pH 7.4, we were expecting a mixture of paramagnetic and diamagnetic species in equilibrium (NiH_2_L, NiHL, NiL) and hence only a 0.1 equivalent of Ni(II) ions was used. As in the case of Zn(II) ions, broadening may be a result not of a paramagnetic effect, but of the dynamics and fast exchange of the system.^37–39^ The loss of the next three protons leads to the formation of the NiH–_1_L, NiH–_2_L and NiH–_3_L forms; all three deprotonations correspond to the binding of three subsequent amide nitrogens (Tables 1 and 2). CD spectra confirm this finding with pronounced bands with a negative Cotton effect at 445 nm and a positive Cotton effect at 525 nm (Supplementary Fig. S7D, ESI†), which are typical of amide involving square–planar nickel–peptide complexes.

**Fig. 6.**
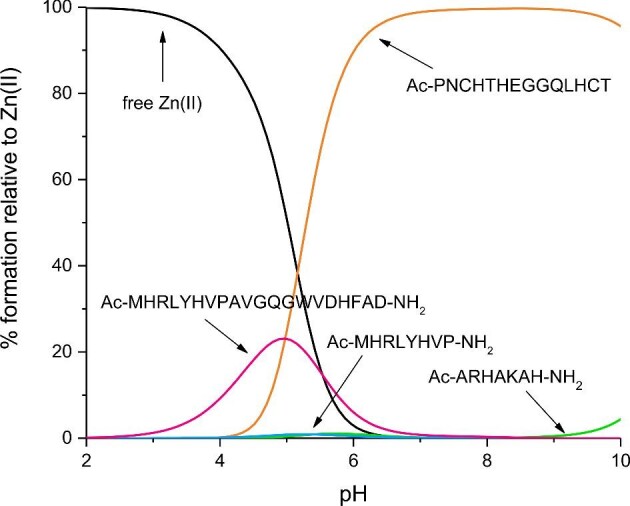
A competition plot between Aspf2 fragments: Ac–ARHAKAH–NH_2_, Ac–MHRLYHVP–NH_2_, Ac–MHRLYHVPAVGQGWVDHFAD–NH_2_, Ac–PNCHTHEGGQLHCT, and Zn(II), describes complex formation at different pH values in a hypothetical situation in which equimolar amounts of all reagents are mixed. Calculations are based on binding constants from Table 1. Conditions: 298 K, I = 0.1 M, [Zn(II)] = [Ac–ARHAKAH–NH_2_] = [Ac–MHRLYHVP–NH_2_] = [Ac–MHRLYHVPAVGQGWVDHFAD–NH_2_] = [Ac–PNCHTHEGGQLHCT] = 0.001 M.

In order to establish which Aspf2 zincophore region binds Zn(II) with the highest affinity, basing on the potentiometric data, we simulated a hypothetical situation, in which equimolar amounts of Zn(II) and all studied Aspf2 regions are mixed—this approach allows a direct comparison of the calculated constants at different pH values (Fig. 6). Above pH 6, the Ac–PNCHTHEGGQLHCT C-terminal Aspf2 fragment becomes the primary zinc-binding site. At around physiological pH (pH 7.4), more than 95% of metal is coordinated to this sequence, being bound to two histidine imidazoles and two cysteine thiolates. The participation in the binding of Zn(II) by the remaining analysed sequences (Ac–ARHAKAH–NH_2_, Ac–MHRLYHVP–NH_2_ and Ac–MHRLYHVPAVGQGWVDHFAD–NH_2_) is quite small. The differences in the binding affinity of Zn(II) are due to differences in the coordination modes of these ions.

At acidic pH, the Asp residue present in the Ac–MHRLYHVPAVGQGWVDHFAD–NH_2_ peptide becomes the anchoring site for Zn(II) (its pK_a_ is lower than the pK_a_ of Glu in the Ac–PNCHTHEGGQLHCT peptide). In our hypothetical simulation, at pH 5, about 23% of the available Zn(II) would be bound to Ac–MHRLYHVPAVGQGWVDHFAD–NH_2_, but at pH 7.4, less than 1% of this metal would choose to bind this sequence (at physiological pH, 3 His imidazoles and one carboxylic side chain are the Zn(II)-binding sites; Table 2). The highest affinity of Zn(II) to the Ac–PNCHTHEGGQLHCT peptide fragment is due to the coordination mode similar to that of zinc fingers, where two His imidazoles and two Cys thiolates participate in the metal binding.

The thermodynamically most stable Zn(II)-binding site in Pra1, a zincophore from *C. albicans*, is also situated in its evolutionally conserved C-terminal region; however, in this case, the Zn(II) coordination environment consists of four His imidazoles.^15^ This candidal zincophore is able to pass the metal over to the Zrt1 zinc transporter,^6^ and most likely, the C-terminal part of Pra1 can be selectively recognized by Zrt1, which gives hope that the Pra1 C-terminus could be used as a specific targeting molecule to enable precise drug delivery when linked to standard therapeutics or to antimicrobial peptides.^40–42^

In comparison with the Zn(II)-binding Pra1 zincophore region from *C. albicans*, the *A. fumigatus* C-terminal Aspf2 region has a much higher affinity for Zn(II); at pH 6, 60%, and at pH 7.4—more than 80% of this metal would be bound to this sequence in a hypothetical situation, in which equimolar amounts of Zn(II) and both C-termini would be present (Supplementary Fig. S9, ESI†). The comparison of these stabilities also shows that the {2N_im_, 2S^−^} binding mode present in the Ac–PNCHTHEGGQLHCT fragment from *A. fumigatus* is more stable than the {4N_im_} one detected in the Ac–SHCHTHADGEVHC zincophore region from *C. albicans*.

The interactions of this C-terminal biologically crucial region of the Aspf2 zincophore, responsible for zinc coordination, with ZrfC, the *A. fumigatus* zinc transporter, will be the subject of further studies.

The Aspf2 region, which binds Ni(II) with the highest affinity, is, as in the case of Zn(II), the C-terminal part of the zincophore, Ac–PNCHTHEGGQLHCT. At pH 7.4, the coordination of the nickel ions by Ac–PNCHTHEGGQLHCT involves two histidine imidazoles and two cysteine thiolates (Fig. 5). At basic pH, the nickel coordination mode to Ac–PNCHTHEGGQLHCT changes, most likely to a {3N^–^, S^–^} one, with the C-terminal part of this zincophore remaining the strongest Ni(II)-binding site. The strength of nickel binding may also be influenced by the secondary structure of the peptide. Interestingly, at pH 7.4, Ni(II) binding to Ac–PNCHTHEGGQLHCT induces the formation of a partial poly proline II (PPII) like structure (Supplementary Fig. S10A, ESI†), while the structures of this complex at other pH values and the structures of its Zn(II) complex are not well defined (Supplementary Fig. S10B, ESI†), although at pH 7.4, both Zn(II) and Ni(II) occupy the same binding site. NMR results also suggest differences in the folding of the Zn(II) and Ni(II) peptide complexes—we observe differences in the position of signals for the residues remote from the binding site, such as on glutamine H_α_–NH correlations, which may further suggest differences in the folding of the peptide complexes.

**Fig. 7.**
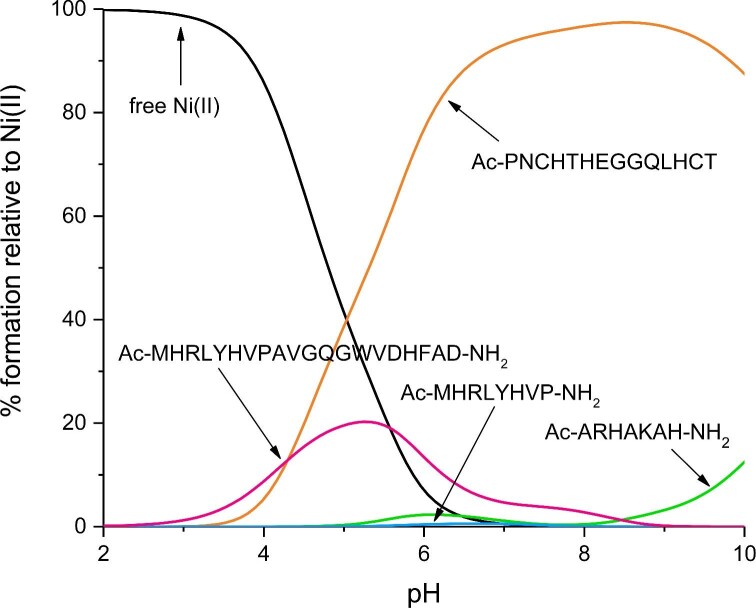
A competition plot between Aspf2 fragments: Ac–ARHAKAH–NH_2_; Ac–MHRLYHVP–NH2, Ac–MHRLYHVPAVGQGWVDHFAD–NH_2_, Ac–PNCHTHEGGQLHCT and Ni(II), describes complex formation at different pH values in a hypothetical situation in which equimolar amounts of all reagents are mixed. Calculations are based on binding constants from Table 1. Conditions: 298 K, I = 0.1 M, [Ni(II)] = [Ac–ARHAKAH–NH_2_] = [Ac–MHRLYHVP–NH_2_] = [Ac–MHRLYHVPAVGQGWVDHFAD–NH_2_] = [Ac–PNCHTHEGGQLHCT] = 0.001 M.

The remaining three Aspf2 fragments analysed in this work do not show high affinity for Ni(II). At acidic pH, the Ac–MHRLYHVPAVGQGWVDHFAD–NH_2_ fragment with one aspartic acid residue, binds Ni(II) with the highest affinity, most likely due to the presence of the Asp residue. The shorter Aspf2 regions (Ac–ARHAKAH–NH_2_ and Ac–MHRLYHVP–NH_2_) have an even lower affinity toward Ni(II) ions (Fig. 7).

**Fig. 8.**
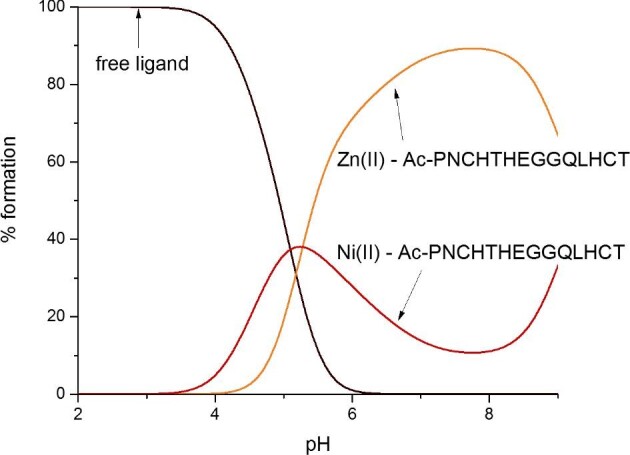
Competition plot between Aspf2 fragment Ac–PNCHTHEGGQLHCT, Zn(II) and Ni(II), describes complex formation at different pH values in a hypothetical situation in which equimolar amounts of the three reagents are mixed. Calculations are based on binding constants from Table 1.

**Fig. 9.**
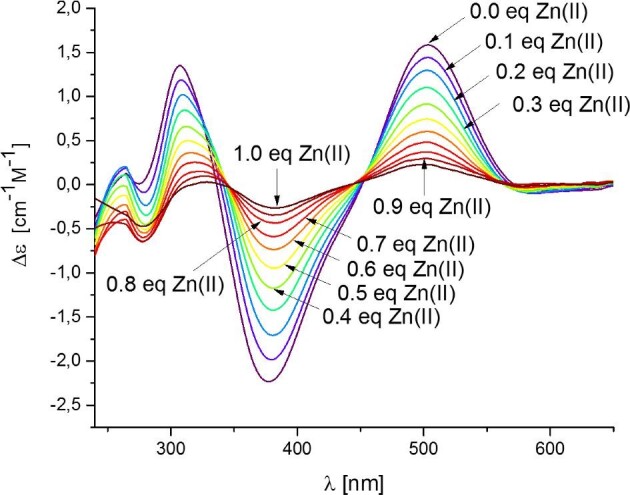
CD spectra of Ni(II) complexes of the Aspf2 protein fragment (Ac–PNCHTHEGGQLHCT) at the pH 7.4, titrated with Zn(II) ions at a step of 0.1 molar equivalents. Final conditions: [Zn(II)]: [Ni(II)]: [Ac–PNCHTHEGGQLHCT] = 1:1:1. Spectra were recorded at 298 K, in the range 240–650 nm and with the optical path 1 cm.

The Aspf2 zincophore strongly prefers to bind Zn(II) over Ni(II); even in a theoretical situation in which equimolar amounts of both metals would be available, at pH 7.4, about 85% of the C-terminal part of the zincophore would bind Zn(II) (Fig. 8). From the biological point of view, this suggests a Zn(II) specificity of Aspf2; from the point of view of coordination chemistry, it shows that the {2N_im_, 2S^−^} binding mode, typical for zinc fingers, is indeed more tempting for Zn(II) than for Ni(II) ions.^43,44^ To additionally prove which metal ion is more strongly bound to the C-terminal fragment of the Aspf2 protein, we performed a CD-spectroscopic titration of the Ni(II)–Ac–PNCHTHEGGQLHCT species with Zn(II) at pH 7.4 with a step of 0.1 molar equivalents and observed a stepwise displacement of Ni(II) with Zn(II) ions [naturally, for Zn(II), a d^10^ metal, no d–d bands are visible] (Fig. 9), which further confirms that Zn(II) is able to displace Ni(II) from its binding site.

What about the other potential metal-binding sites? In a theoretical situation in which equimolar amounts of Zn(II) and Ni(II) would be available for Ac–ARHAKAH–NH_2_, Ac–MHRLYHVP–NH_2_ and Ac–MHRLYHVPAVGQGWVDHFAD–NH_2_ peptide fragments at pH 7.4, all sequences would bind Ni(II) with higher affinity than Zn(II) (Supplementary Fig. S11, ESI†) (however with much lower affinities than the C-terminal site). From the chemical point of view, it shows that a binding mode involving histidine residues is more tempting for Ni(II) than for Zn(II) ions. The general lower affinity for both metal ions in these sequences in comparison to the C-terminal one, Ac–PNCHTHEGGQLHCT, is due to the absence of cysteine residues in the analysed fragments, because the cysteine residues play an important role as anchoring groups for Zn(II) and Ni(II), with which they form very stable complexes with binding modes similar to that of zinc fingers. The obtained results are in agreement with what could have maybe been expected from the Irving–Williams series—experiments carried out with small chelating agents show that the HS–CH_2_–CH_2_–NH_2_ ligand binds Zn(II) a little stronger than Ni(II), while the H_2_N–CH_2_–CH_2_–NH_2_ one has a stronger affinity toward Ni(II).^45^ Taken together, the data show that the mixture of thiolates and amines shows good selectivity for Zn(II) ions.

The low affinity of the Ac–ARHAKAH–NH_2_, Ac–MHRLYHVP–NH_2_ and Ac–MHRLYHVPAVGQGWVDHFAD–NH_2_ sequences for Zn(II) in the whole pH range indicates that there is a low probability that Aspf2 bind the two metal ions in different sites. Additionally, competition plots comparing the binding strength of Zn(II) and Ni(II) for the remaining sequences indicate that at physiological pH, Zn(II) are bound with a very low affinity compared to Ni(II). However, after binding the metal ion in the primary binding site, the conformation of the native protein may slightly change and the availability of the other binding sites may differ from the affinity obtained for model sequences.

In order to be able to compare the obtained results with the affinities of other metal-binding peptides, potential competing proteins and other biomolecules we have calculated conditional K_D_ values at pH 7.4 for all studied complexes (Supplementary Table S1, ESI†). They further confirm that both Zn(II) and Ni(II)–Ac–PNCHTHEGGQLHCT complexes are the most stable ones (with pK_D_ values of 8.83 and 7.03 for Zn(II) and Ni(II), respectively; the constants for the other ligands have lower values, in the range of 3.39–5.66).

## Conclusions

Zinc is crucial for the virulence and survival of *A. fumigatus* in the host organism. This work is an input to the basic chemistry of Zn(II) and Ni(II) ions, which allows us to understand the bioinorganic chemistry of fungal zincophores. Four potential *A. fumigatus* Aspf2 zincophore regions have been characterized using mass spectrometry, potentiometry, UV–Vis, CD and NMR spectroscopy, showing that the most probable Zn(II)-binding site on the Aspf2 zincophore is its C-terminal region, Ac–PNCHTHEGGQLHCT. Moreover, at pH 7.4, the same C-terminal part of the zincophore binds Ni(II) via an analogous coordination mode {2N_im_, 2S^–^}, but with a smaller affinity that Zn(II). The PNCHTHEGGQLHCT region of the zincophore may probably serve as a targeting molecule that is able to (analogously to the *C. albicans* Pra1 zincophore) bring an appropriately bound therapeutic to the close proximity of *A. fumigatus*, specifically passing over the Zn(II) ion to the ZrfC transporter.

## Supplementary Material

mfac042_Online_Appendix

## Data Availability

Data available on request.
