# Supplementary material for: Specific Zn(II)-binding site in the C-terminus of Aspf2, a zincophore from Aspergillus fumigatus
Source: Metallomics. 2022 Jun 14;14(7):mfac042. doi: 10.1093/mtomcs/mfac042 (PMC9780748; doi:10.1093/mtomcs/mfac042)
Supplement: mfac042_Online_Appendix [file mfac042_online_appendix.docx]

**Supplementary Information**

Specific Zn(II) binding site in the C-terminus of Aspf2, a zincophore form *Aspergillus fumigatus*

Kinga Garstka,^a^ Aleksandra Hecel,^a^ Henryk Kozłowski^a,b^ and Magdalena Rowińska-Żyrek*^a^

1. Faculty of Chemistry, University of Wrocław, F. Joliot-Curie 14, 50-383 Wrocław, Poland. E-mail: magdalena.rowinska-zyrek@chem.uni.wroc.pl
2. Institute of Health Sciences, University of Opole, Katowicka 68 St, 45-060 Opole, Poland.

The detected MS signals correspond to equimolar Zn(II) complexes (*m/z*= 447.19, *z*=2+, *m/z*=578.24, *z*=2+, *m/z*=813.02, *z*=3+, *m/z*=818.28, *z*=2+ for the Ac-ARHAKAH-NH_2_, Ac-MHRLYHVP-NH_2_, and Ac-MHRLYHVPAVGQGWVDHFAD-NH_2_, Ac-PNCHTHEGGQLHCT, respectively). Experimental peak assignment is compared to simulated isotopic patterns, witch fit perfectly with the experimental ones. In all measured mass spectra, signals corresponding to sodium adducts of the ligands are observed. (Fig. S1-2, ESI†).

In the case of Zn(II)-Ac-ARHAKAH-NH_2_ mass spectra (Fig. S1A, ESI†) the prevailing signals correspond to the free ligand (*m/z*=831.46, *z*=1+ and *m/z*=416.23, *z*=2+), and sodium adducts of the peptide: with one sodium atom (*m/z*= 853.44, *z*=1+ and *m/z*=427.23, *z*=2+) and two sodium atoms (*m/z*=438.22, *z*=2+). Signals which correspond to zinc complex sodium adducts are also visible (*m/z*=916.43, *z*=1+ and *m/z*= 458.18, *z*=2+).

In the Zn(II)-Ac-MHRLYHVP-NH_2_ mass spectra (Fig. 1B, ESI†), aside from the signals with come from the free ligand (*m/z*=1093.55 , *z*=1+, *m/z*=547.28, *z*=2+ and *m/z*=365.19, *z*=3+), a sodium adduct (*m/z*= 558.28, *z*=2+) and a zinc complexes sodium adduct (*m/z*=589.23, *z*=2+) is visible.

In the Zn(II)-Ac-MHRLYHVPAVGQGWVDHFAD-NH_2_ mass spectra, signals which come from the free ligand (*m/z*=1189.05, *z*=2+ and *m/z*=793.04, *z*=3+) sodium adducts with one (*m/z*=1200.04, *z*=2+ and *m/z*=800.37, *z*= 3+ and *m/z*=600.53, *z*=4+), two (*m/z*=1211.04, *z*=2+, *m/z*=807.70, *z*=3+ and *m/z*=606.03, *z*=4+) and three sodium atoms (*m/z*=815.03, *z*=3+ and *m/z*=611.52, *z*=4+) and a zinc complex with one sodium atom (*m/z*= 813.02, *z*= 3+) are visible (Fig. S1C, ESI†).

In the Zn(II)-Ac-PNCHTHEGGQLHCT mass spectra (Fig. S1D, ESI†), beside from the signal which comes from the free ligand (*m/z*=788.31, *z*=2+), sodium adducts of the ligand with one (*m/z*= 799.30, *z*= 2+ and *m/z*=533.21, *z*=3+), two (*m/z*=810.29, *z*=2+ and *m/z*=540.53, *z*=3+), three (*m/z*=821.29, *z*=2+ and *m/z*=547.86, *z*=3+) and four sodium atoms (*m/z*=555.19, *z*=3+) are visible. Signal which corresponds to the zinc complex is visible at *m/z*=818.28 (*z*=2+).

In the Ni(II)-Ac-ARHAKAH-NH_2_ spectra (Fig. S2A, ESI†), the signals with come from the free ligand (*m/z*=831.45, *z*=1+ and *m/z*=416.23, *z*=2+), its sodium adduct with one (*m/z*=853.44, *z*=1+) and two sodium atoms (*m/z*=438.22, *z*=2+), and nickel complex (*m/z*=887.37, *z*=1+ and *m/z*=444.19, *z*=2+) and its sodium adducts (*m/z*=455.19, *z*=2+) are observed.

In the Ni(II)-Ac-MHRLYHVP-NH_2_ mass spectra (Fig. S2B, ESI†), beside the signals from the ligand (*m/z*=1093.55, *z*=1+, *m/z*= 547.28, *z*=2+ and *m/z*=365.19, *z*=3+), sodium adducts of the ligand with one (*m/z*=558.28, *z*=2+) and two sodium atoms (*m/z*=569.27, *z*=2+), a nickel complex (*m/z*=575.24, *z*=2+) are visible.

In the case of Ni(II)-Ac-MHRLYHVPAVGQGWVDHFAD-NH_2_ (Fig. S2C, ESI†) the prevailing signal comes from the free ligand (*m/z*=793.09, *z*=3+). There is also a signal at *m/z*=1189.05 (*z*=2+) and the signals from its sodium adducts with one (*m/z*=1200.04, *z*=2+, *m/z*=800.37, *z*=3+ and *m/z*=600.53, *z*=4+) and two sodium atoms (*m/z*=807.70, *z*=3+ and *m/z*=606.03, *z*=4+); a nickel complex (*m/z*=811.68, *z*=3+ and *m/z*=609.02, *z*=4+) and its sodium adducts (*m/z*=819.01, *z*=3+ and *m/z*=614.51, *z*=4+) are also observed. The simulated isotopic patterns of all complexes fit perfectly with the experimental ones.

In the Ni(II)-Ac-PNCHTHEGGQLHCT spectra (Fig. S2D, ESI†), aside from the signal witch comes the free ligand (*m/z*=788.31, *z*=2+ and *m/z*=525.88, *z*=3+), a sodium adduct with one sodium atom (*m/z*=799.30, *z*=2+ and *m/z*=531.21, *z*=3+) and two sodium atoms (*m/z*=540.54, *z*=3+) and a nickel complex (*m/z*=816.27, *z*=2+ and *m/z*=544.52, *z*=3+) can be observed. All simulated isotopic patterns fit perfectly with the simulated ones.


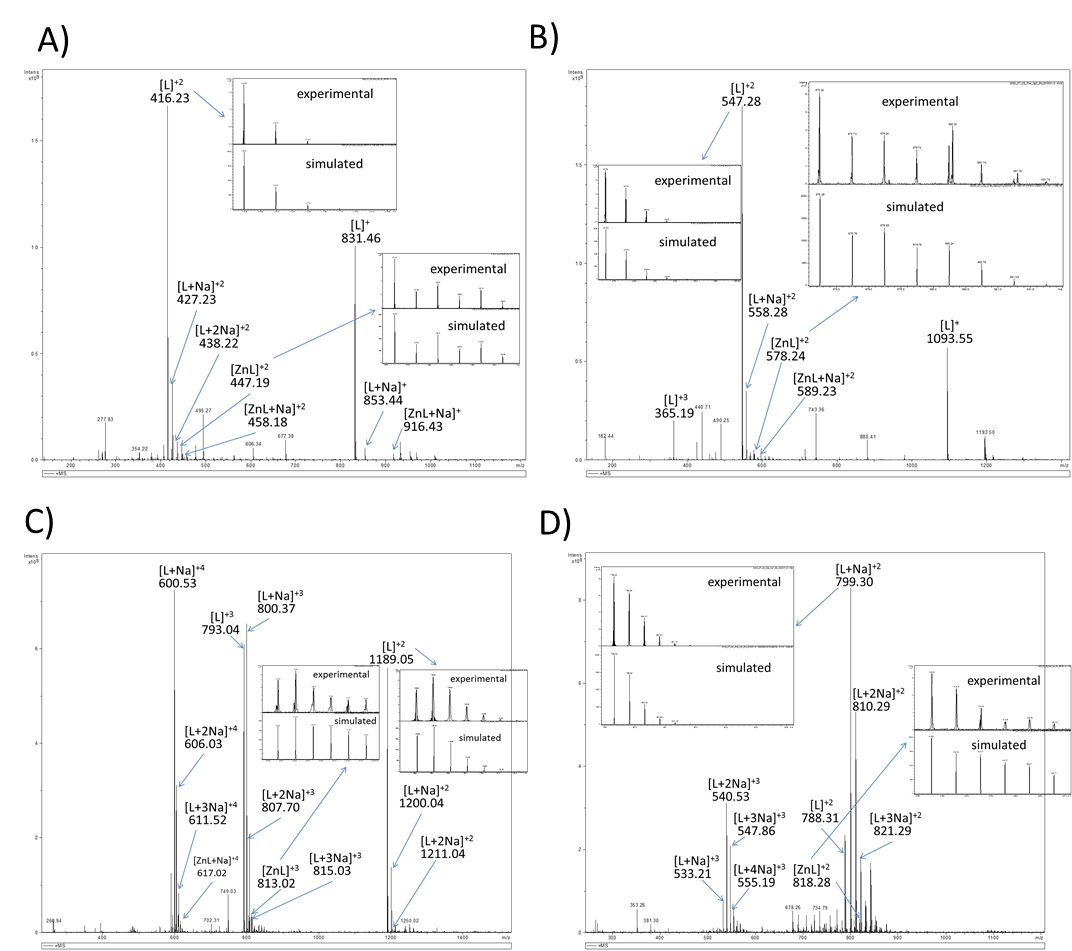
Figure 1. ESI-MS spectrum of A) Zn(II)-Ac-ARHAKAH-NH_2_ ; B) Zn(II)-Ac-MHRLYHVP-NH_2_; C) Zn(II)-Ac- MHRLYHVPAVGQGWVDHFAD-NH_2_, D) Zn(II)-Ac-PNCHTHEGGQLHCT; Zn(II)/L molar ratio=1:1.


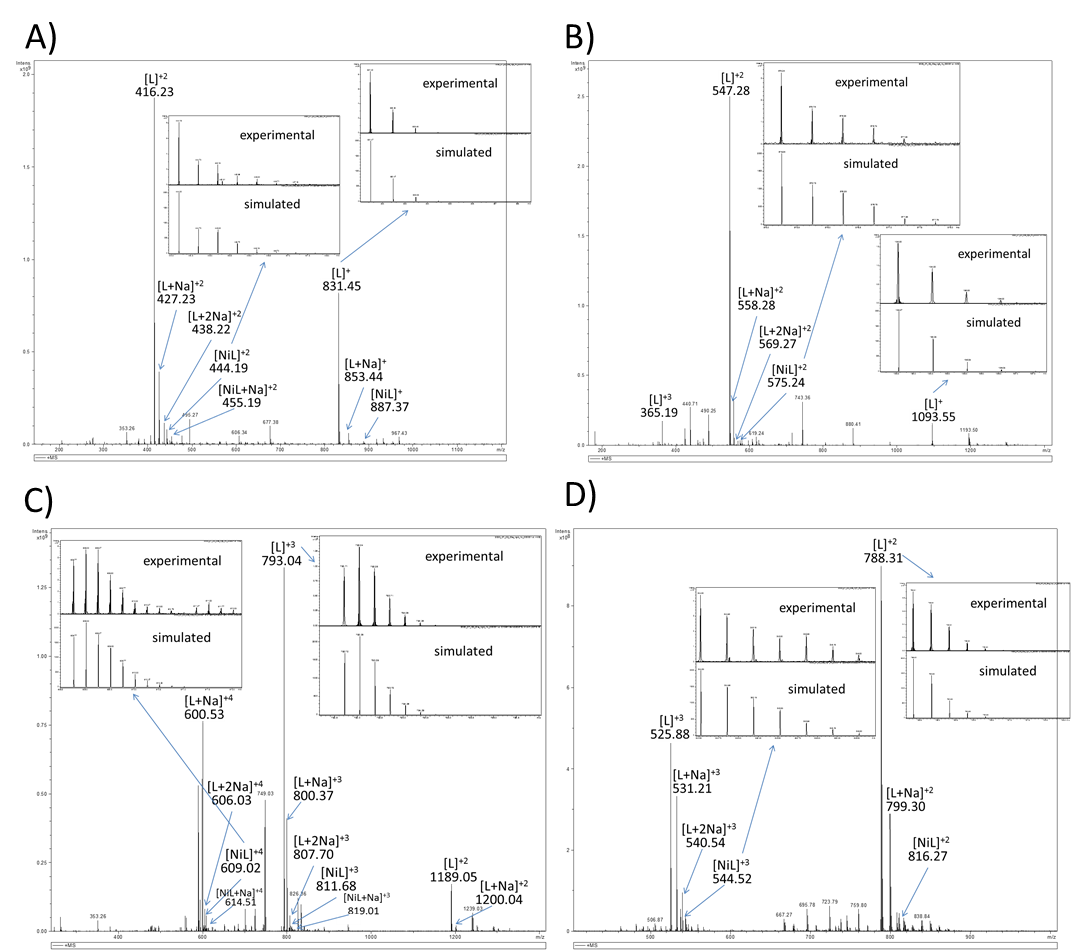
Figure 2. ESI-MS spectrum of A) Ni(II)-Ac-ARHAKAH-NH_2_; B) Ni(II)-Ac-MHRLYHVP-NH_2_; C) Ni(II)-Ac- MHRLYHVPAVGQGWVDHFAD-NH_2_, D) Ni(II)-Ac-PNCHTHEGGQLHCT; Ni(II)/L molar ratio=1:1.


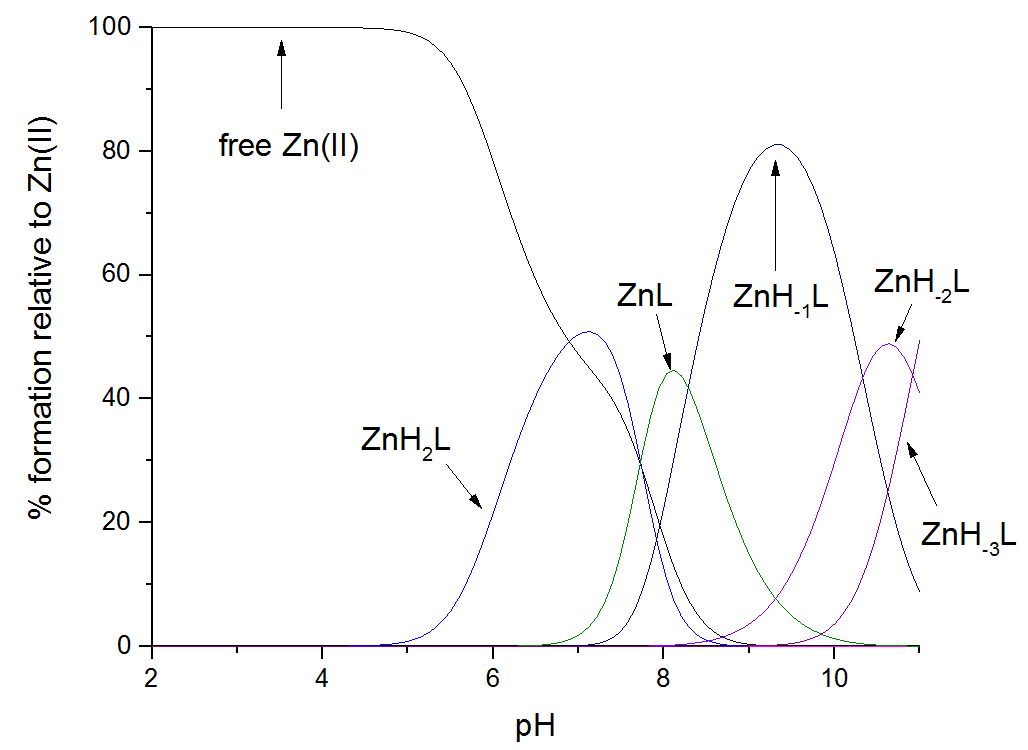

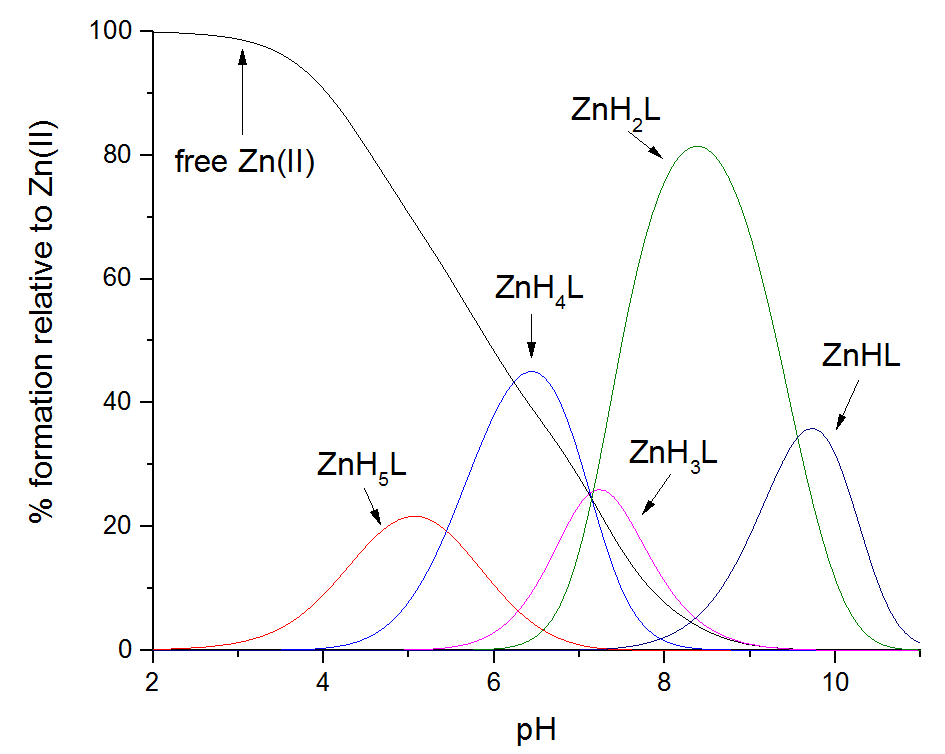
Figure 3. Distribution diagrams for the formation of A) Zn(II) complex with the Ac-ARHAKAH-NH_2_ Aspf2 fragment; B) Zn(II) complex with the Ac-MHRLYHVP-NH_2_ Aspf2 fragment; C) Zn(II) complexes with Ac-MHRLYHVPAVGQGWVDHFAD-NH_2_ Aspf2 fragment; D) Zn(II) complex with the Ac-PNCHTHEGGQLHCT Aspf2 fragment. Zn(II)/L molar ratio=1:1.


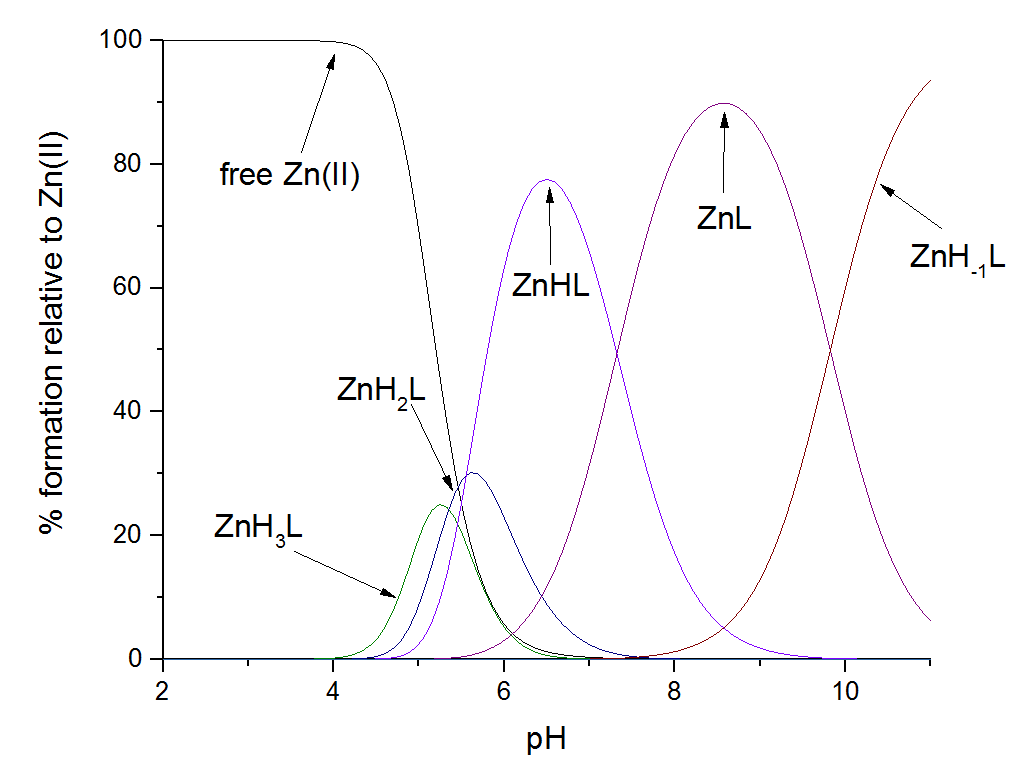

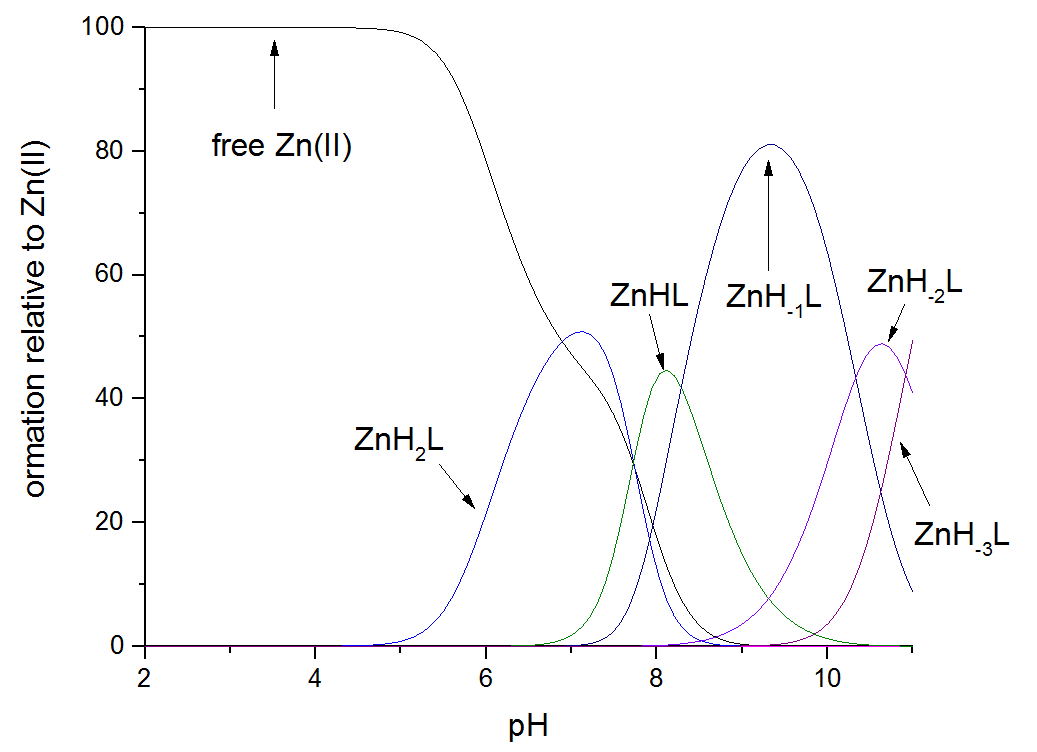

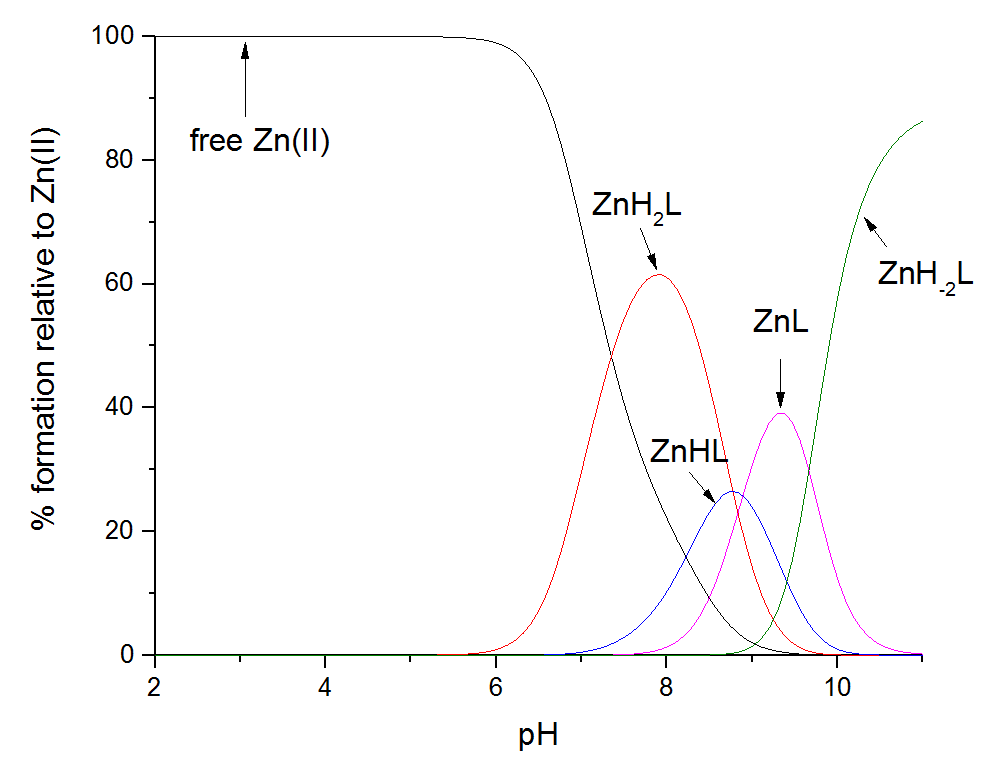

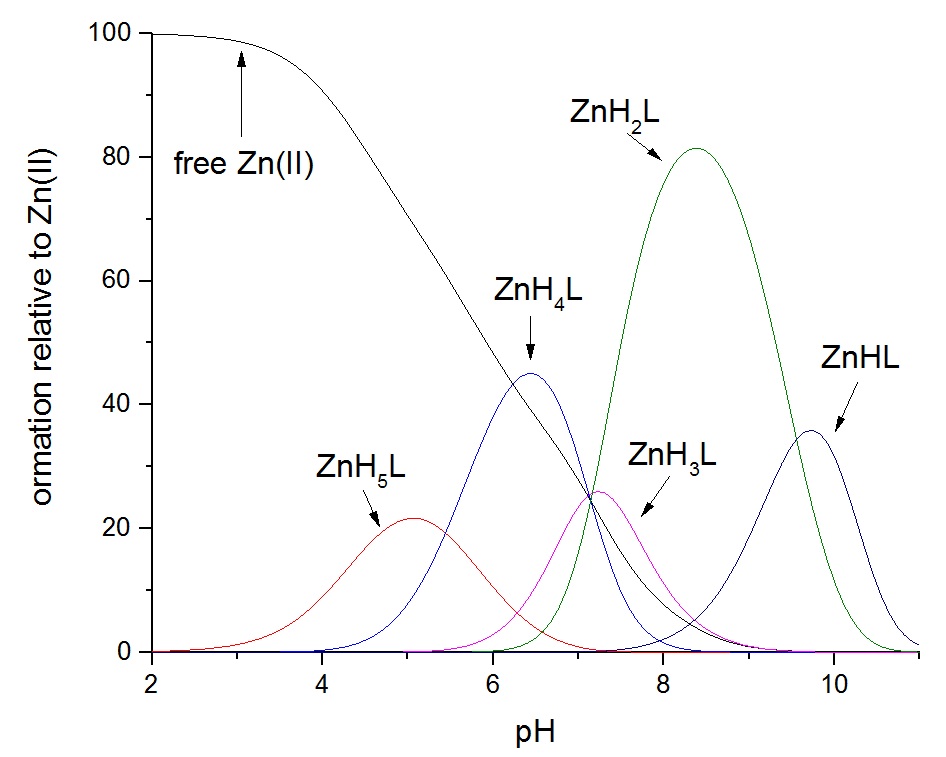


A)

B)

C)

D)


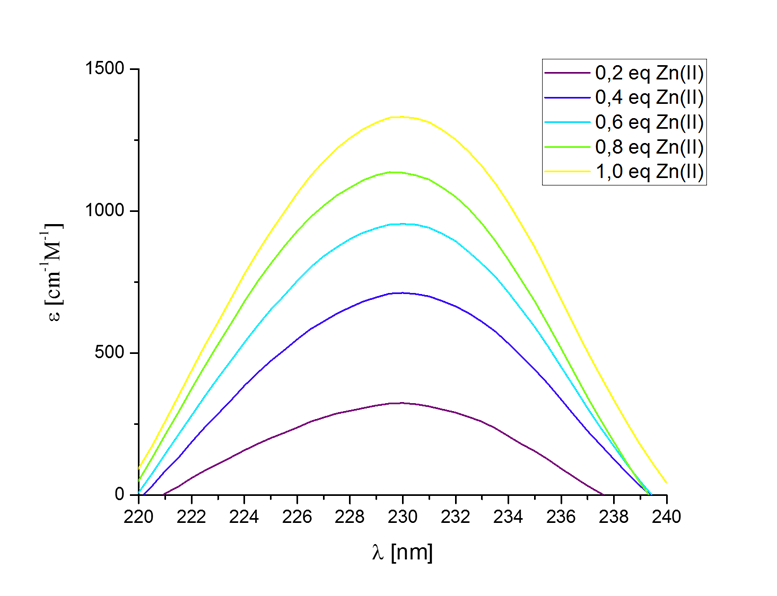


Figure 4. UV-Vis spectra of Zn(II) complexes of the Aspf2 protein fragment (Ac-PNCHTHEGGQLHCT) at the pH 7.4, titrated with Zn(II) ions at a step of 0.2 molar equivalents. Final conditions: [Zn(II)]:[Ac-PNCHTHEGGQLHCT]=1:1. Spectra were recorded at 298 K, in the range 220-240 nm and with the optical path 0.1 cm.


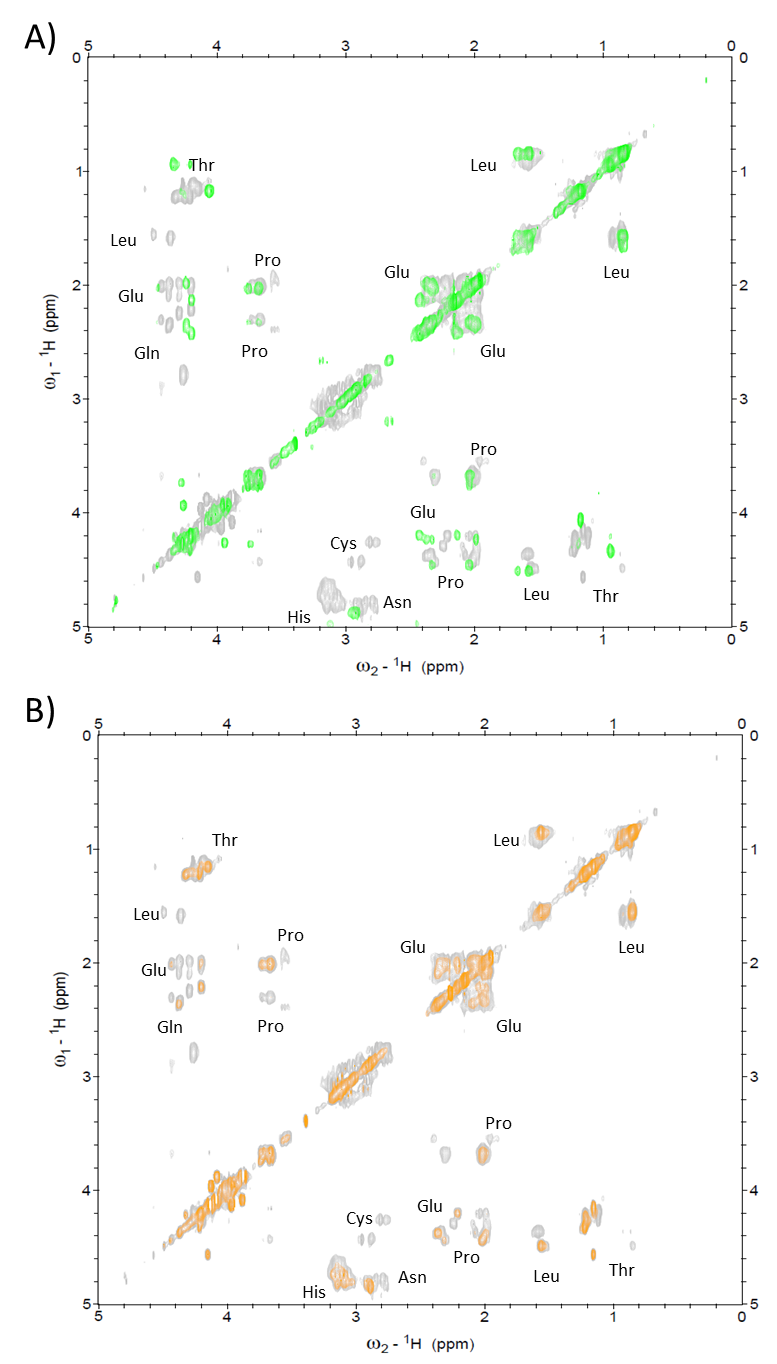


Figure 5. TOCSY spectra of 3 mM Ac-PNCHTHEGGQLHCT Aspf2 fragment pH 7.4, in the absence (gray) and presence of A) 1 Zn(II) equivalent (green); B) 0.1 Ni(II) equivalents (orange).

Figure 6. Distribution diagrams for the formation of: A) Ni(II) complex with the Ac-ARHAKAH-NH_2_ Aspf2 fragment; B) Ni(II) complex with the Ac-MHRLYHVP-NH_2_ Aspf2 fragment; C) Ni(II) complexes with Ac-MHRLYHVPAVGQGWVDHFAD-NH_2_ Aspf2 fragment; D) Ac-PNCHTHEGGQLHCT Aspf2 fragment. Ni(II)/L molar ratio=1:1.


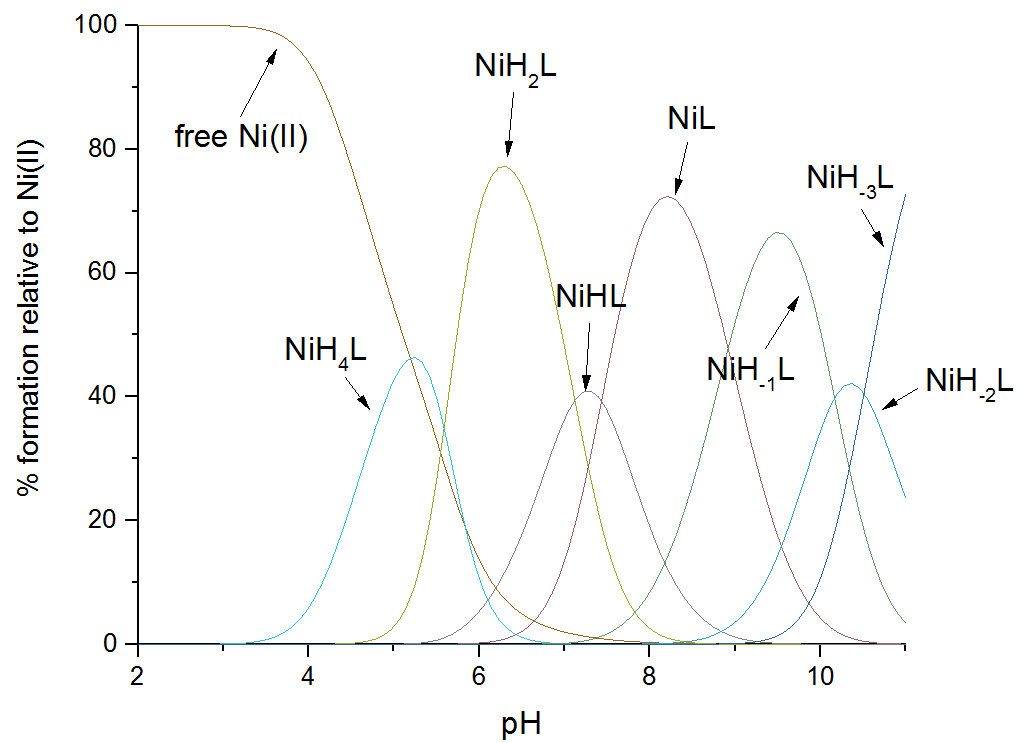

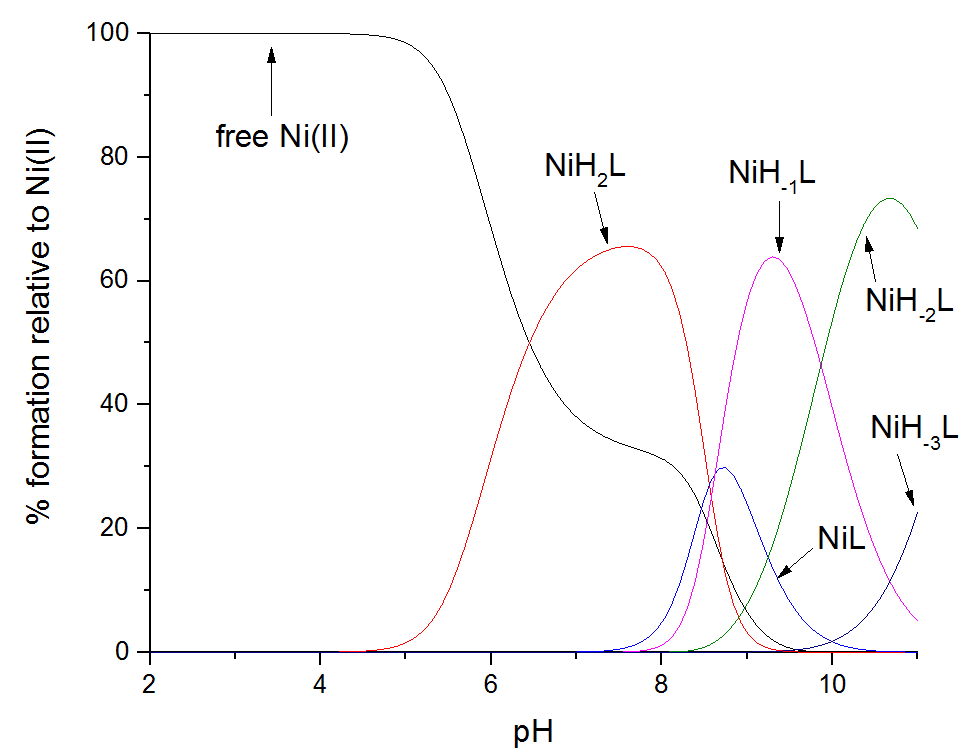

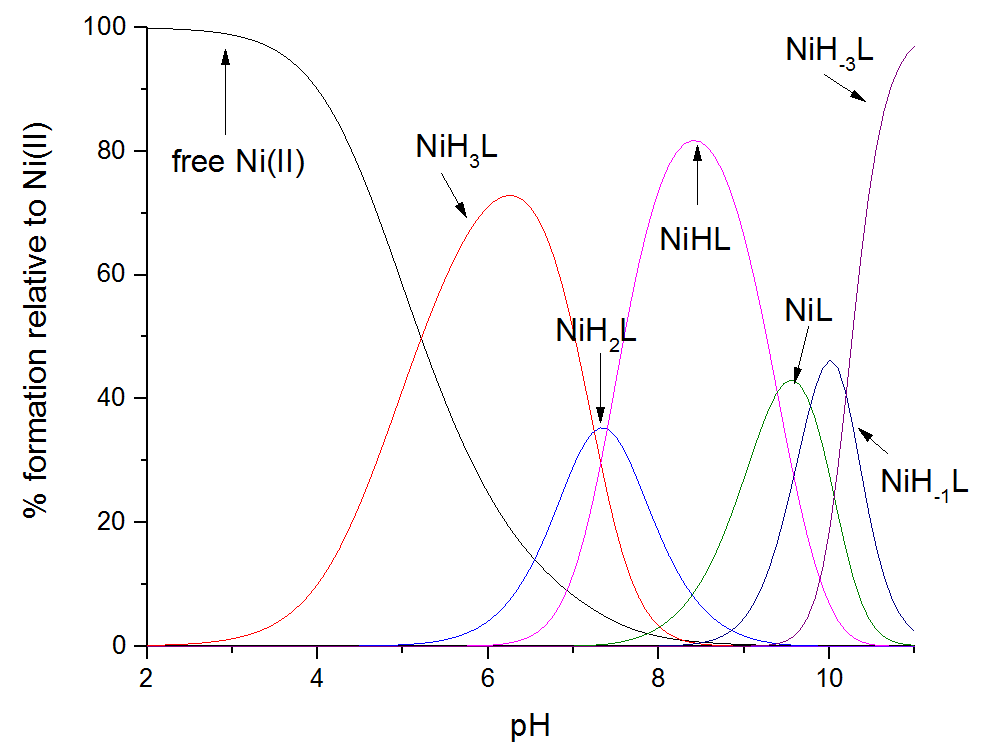

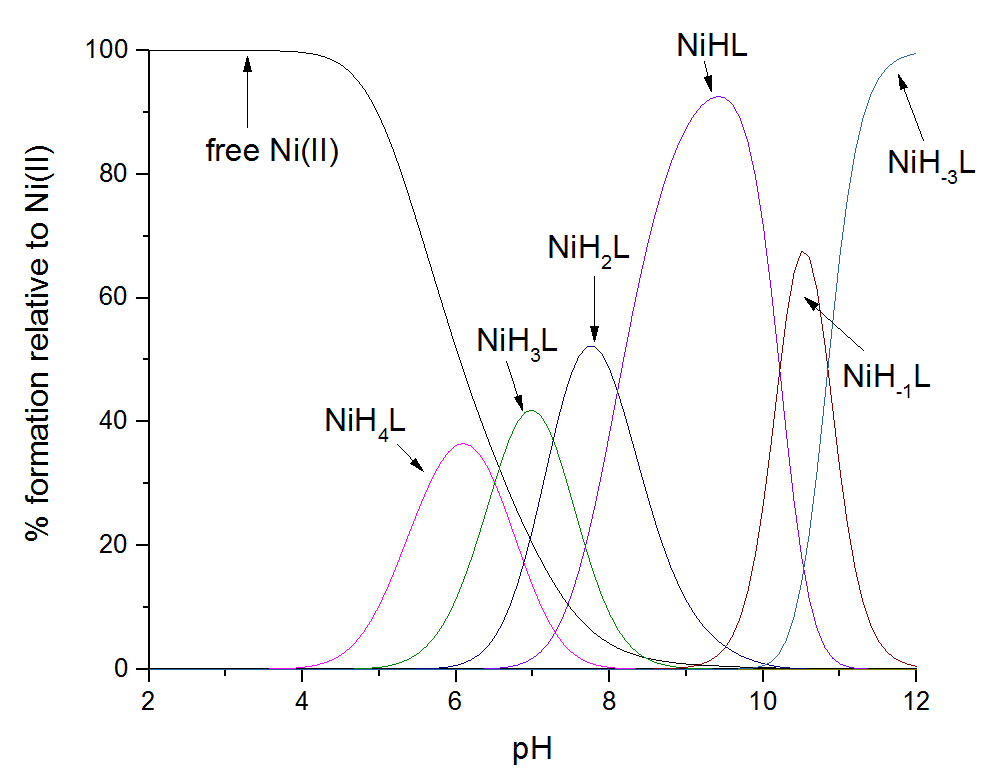


A)

B)

C)

D)

Figure 7. CD spectra of Ni(II) complexes with A) Ac-ARHAKAH-NH_2_; B) Ac-MHRLYHVP-NH_2_; C) Ac-MHRLYHVPAVGQGWVDHFAD-NH_2_; D) Ac-PNCHTHEGGQLHCT. Optical path 1 cm, Ni(II)/L molar ratio=1:1.

A)

B)

C)

D)

Figure 8. UV-Vis spectra of Ni(II) complexes with A) Ac-ARHAKAH-NH_2_; B) Ac-MHRLYHVP-NH_2_; C) Ac-MHRLYHVPAVGQGWVDHFAD-NH_2_; D) Ac-PNCHTHEGGQLHCT; Ni(II)/L molar ratio=1:1.

A)

B)

C)

D)

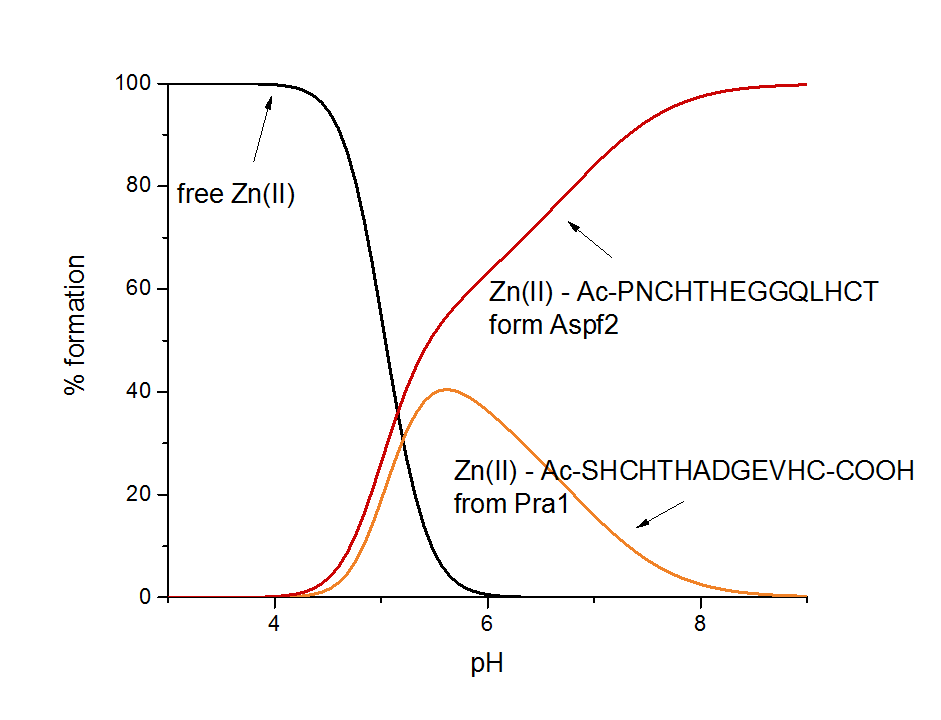
Figure 9. A competition plot between Aspf2 fragment: Ac-PNCHTHEGGQLHCT, Pra1 fragment: Ac-SHCHTHADGEVHC-COOH and Zn(II), describes complex formation at different pH values in a hypothetical situation in which equimolar amounts of all reagents are mixed. Calculations are based on binding constants from Table 1 and [1]. Conditions: 298 K, I=0.1 M, [Zn(II)]=[Ac-PNCHTHEGGQLHCT]=[Ac-SHCHTHADGEVHC-COOH]=0.001 M.

Figure 10. CD spectra of A) Ac-PNCHTHEGGQLHCT complex with Ni(II), PPII - polyproline II helix; B) Ac-PNCHTHEGGQLHCT complex with Zn(II); optical path 0.1 mm, Ni(II)/L molar ratio=1:1.


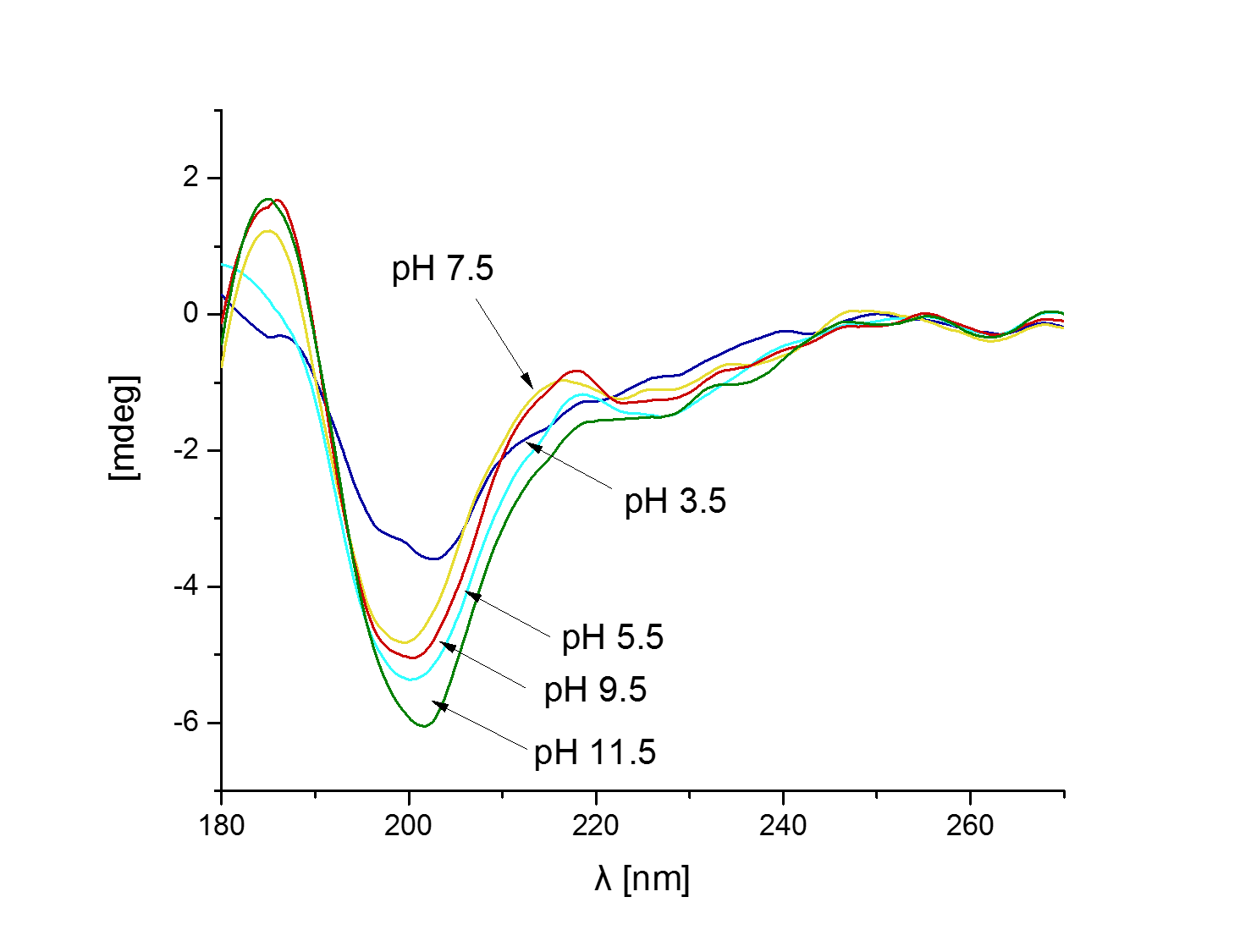


A)

B)


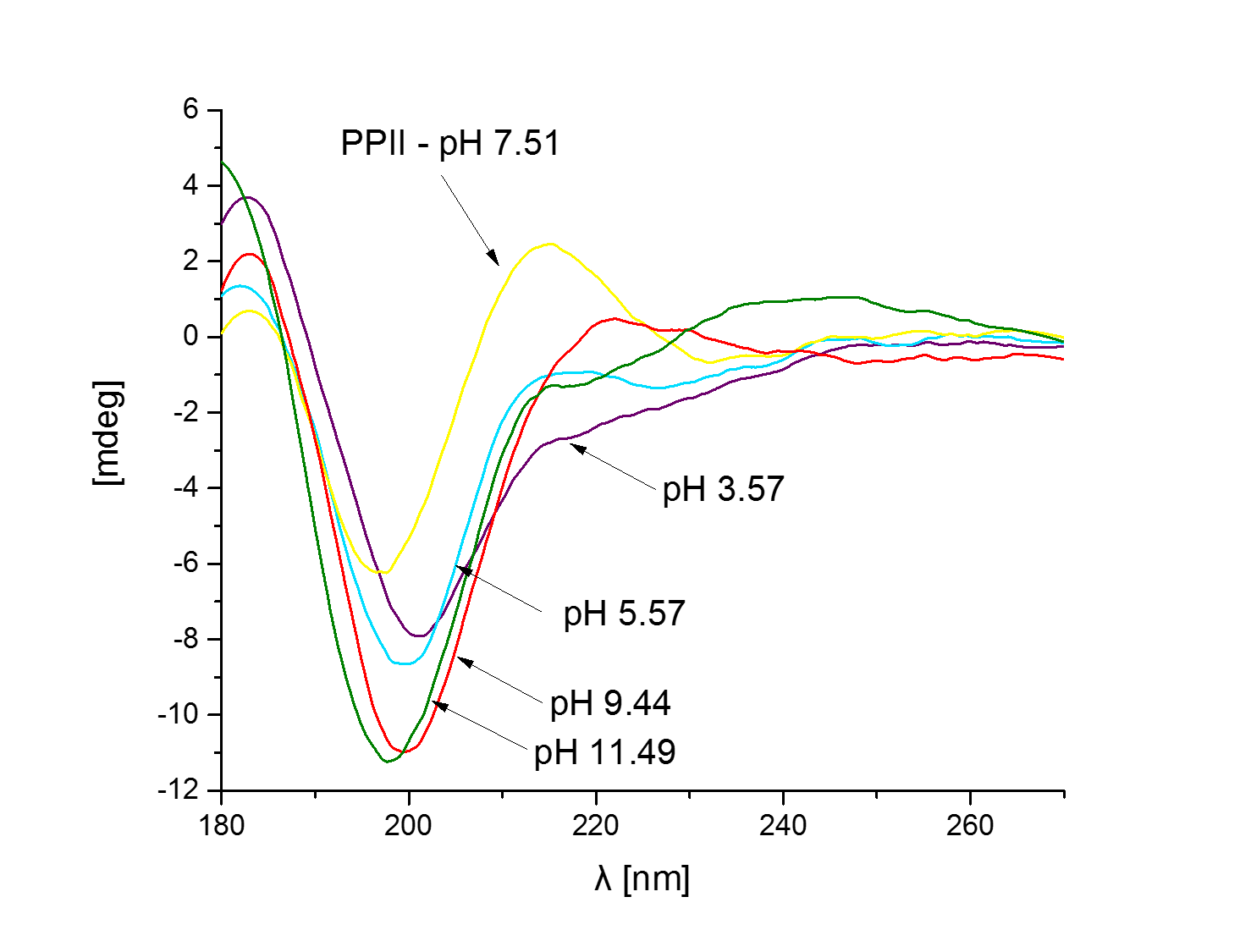


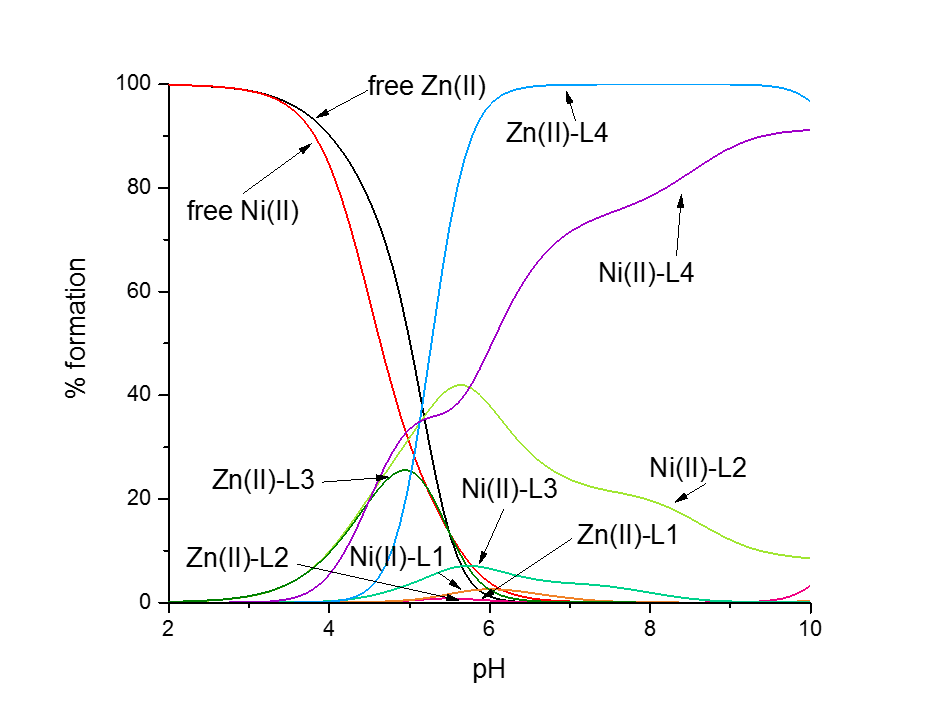
Figure 11. Competition plot between Aspf2 fragments; where: L1 - Ac-ARHAKAH-NH_2_; L2 - Ac-MHRLYHVP-NH_2_; L3 - Ac-MHRLYHVPAVGQG WVDHFAD-NH_2_; L4 - Ac-PNCHTHEGGQLHCT; Zn(II) and Ni(II), describes complex formation at different pH values in a hypothetical situation in which equimolar amounts of all reagents are mixed. Calculations are based on binding constants from Table 1.

Table 1. The conditional K_D_ values for Zn(II) and Ni(II) complexes of the: Ac-ARHAKAH-NH_2_, Ac-MHRLYHVP-NH_2_ , Ac-MHRLYHVPAVGQGWVDHFAD-NH_2_ and Ac-PNCHTHEGGQLHCT

|  | K_D_ | pK_D_ |
| --- | --- | --- |
| Zn(II) - Ac-ARHAKAH-NH_2_ | $3,17*{10}^{-4}$ | 3,50 |
| Ni(II) - Ac-ARHAKAH-NH_2_ | $1,79*{10}^{-4}$ | 3,75 |
| Zn(II) - Ac-MHRLYHVP-NH_2_ | $4,09*{10}^{-4}$ | 3,39 |
| Ni(II) - Ac-MHRLYHVP-NH_2_ | $2,21*{10}^{-6}$ | 5,66 |
| Zn(II) - Ac-MHRLYHVPAVGQGWVDHFAD-NH_2_ | $4,29*{10}^{-5}$ | 4,37 |
| Ni(II) - Ac-MHRLYHVPAVGQGWVDHFAD-NH_2_ | $1,64*{10}^{-5}$ | 4,79 |
| Zn(II) - Ac-PNCHTHEGGQLHCT | $1,47*{10}^{-9}$ | 8,83 |
| Ni(II) - Ac-PNCHTHEGGQLHCT | $9,26*{10}^{-8}$ | 7,03 |

**References**

| 1. D. Łoboda, M. Rowińska-Żyrek, *Dalton Transactions* 2017; **46**:13695-13703 |
| --- |
